# Study on the effect and mechanism of Ershiyiwei Lvronghao concentrated Pills in the treatment of nonalcoholic fatty liver disease

**DOI:** 10.3389/fphar.2025.1725610

**Published:** 2026-01-22

**Authors:** Yexin Wu, Bin Chen, Jintao Long, Mingxin Pai, Zhiyong Xiao, Qun Huang, Kang Li, Bengui Ye

**Affiliations:** 1 Key Laboratory of Drug-Targeting and Drug Delivery System of the Education Ministry, Sichuan Engineering Laboratory for Plant-Sourced Drug and Sichuan Research Center for Drug Precision Industrial Technology, West China School of Pharmacy Sichuan University, Chengdu, China; 2 Medical College of Xizang University, Lhasa, China; 3 Department of Ophthalmology, Hospital of Chengdu University of Traditional Chinese Medicine, Chengdu, China; 4 Key Laboratory of Translational Medicine for Human Adaptation to the High-Altitude of Xizang Autonomous Region, High Altitude Medical Research Institute, Xizang Autonomous Region People’s Hospital, Lhasa, China

**Keywords:** ershiyiwei lvronghao concentrated pills, inflammation, NAFLD, oxidative stress, PPARs

## Abstract

**Background:**

Ershiyiwei Lvronghao Concentrated Pills (ESYWLRHW) is the concentrated pill developed and prepared based on the classical prescription in Xizang: Ershiwuwei Lvronghao Pills (ESWWLRHW). However, its effect on nonalcoholic fatty liver disease (NAFLD) remains unclear. The aim of this study is to clarify the therapeutic effect and mechanism of ESYWLRHW on NAFLD.

**Methods:**

High-Performance Liquid Chromatography-Quadrupole Time of Flight-Mass Spectrometry/Mass Spectrometry (HPLC-Q-TOF-MS/MS) was used to analyze the components of ESYWLRHW. The NAFLD model was established by feeding Sprague-Dawley (SD) rats with high-fat diet (HFD) for ten consecutive weeks, and different doses of ESYWLRHW and positive drugs were given for intervention treatment in the last 6 weeks. The pathological and molecular changes of rats in each group were determined after the whole experimental procedure with pathological sections and ELISA experiments, and the metabolomics, proteomics and transcriptomics were used to investigate the possible mechanism.

**Results:**

In this study, 206 compounds of ESYWLRHW were identified. *In vivo* experiments, ESYWLRHW administration significantly ameliorated the pathological manifestations in NAFLD model rats, including attenuating the abnormal body weight gain, reducing the accumulation of hepatic and peripheral fat, and improving dyslipidemia, liver function, and systemic inflammatory response. Metabolomic analysis further revealed that ESYWLRHW treatment upregulated the levels of specific metabolites, such as 12-keto-leukotriene B4 (12-keto-LTB4), 20-COOH-LTB4, glycocholate (GCA), and dehydroepiandrosterone sulfate (DHEAS). Integrated multi-omics analysis and subsequent verification indicated that the therapeutic effects of ESYWLRHW might be mediated by modulating the expression of Peroxisome Proliferator-Activated Receptors (PPARs), which in turn regulated downstream signaling pathways including nuclear factor erythroid 2-related factor 2 (Nrf2) and the Nuclear Factor-kappa B (NF-κB)/NOD-like receptor family, pyrin domain-containing 3 (NLRP3)/Caspase-1 axis.

**Conclusion:**

In summary, ESYWLRHW can regulate the three metabolic pathways: arachidonic acid metabolism, primary bile acid biosynthesis and steroid hormone biosynthesis, and improve oxidative stress and inflammation in NAFLD rats by regulating the expression of PPARs protein, and ultimately alleviate NAFLD.

## Graphical Abstract

**Figure:**
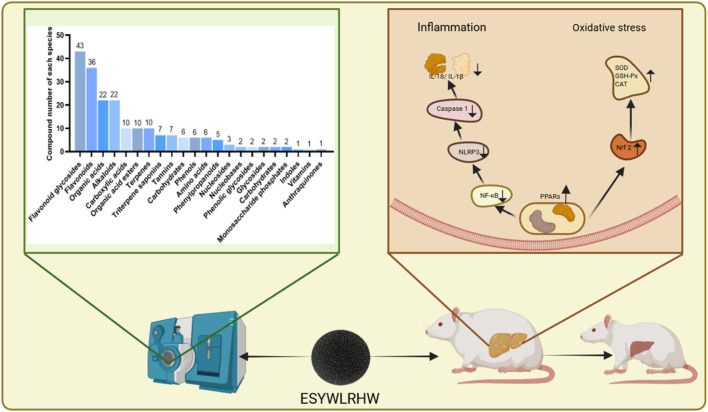

## 1 Introduction

NAFLD was proposed by Ludwig in 1980 to describe the unexplained fatty liver disease in subjects with no history of excessive alcohol consumption (Ludwig et al., 1980). The clinicopathological syndrome of NAFLD is the hepatic lobule as the core area of the lesion, characterized by diffuse bullotic steatosis of hepatocytes and adipose accumulation (Liu and Wang, 2024), which is a popular metabolic liver disease (Duseja et al., 2015; Yki-Järvinen, 2014) and has become the most common chronic liver disease in the world (Riazi et al., 2022; Bessone et al., 2019; Yin et al., 2023), with a prevalence rate of 32.4% in the adult population. And it continues to rise (Riazi et al., 2022; Araújo et al., 2018; Huang et al., 2021), which damage to human health and social economy. With the development of research, the “multiple-hit” theory has been proposed as the most likely explanation for the occurrence and development of NAFLD, which suggests that in addition to triglyceride accumulation and inflammation, other factors, including decreased mitochondrial function, ER stress, oxidative damage and insulin resistance, are also critical factors in NAFLD (Buzzetti et al., 2016; Hardy et al., 2016).

The ESWWLRHW is a classic Xizang medicine prescription, composed of 25 Xizang medicinal materials, such as *Meconopsis quintuplinervia* Regel, *Bambusae Concretio Silicea* and so on, which is mainly used to treat a variety of liver diseases and poisoning symptoms. The prescription has a significant effect of clearing liver and gallbladder, which can effectively relieve the symptoms of liver fever, and has a therapeutic effect on liver tissue lesions such as hepatomegaly and cirrhosis (Commission, 1995; Chen et al., 2009; Basang and Ciren, 2018). In addition, the drug can also improve the pain and discomfort caused by liver and stomach stasis, which is suitable for the treatment of liver disease. And this prescription has a unique regulating effect on “wood cloth” disease and bile organ dysfunction in the theory of Xizang medicine. However, there exists some shortcomings such as simple preparation process, no effective substance extraction, large daily dose and poor patient compliance. Therefore, in this study, we carried out secondary development on ESWWLRHW, 15 medicinal materials including *Meconopsis quintuplinervia* Regel, *Bambusae Concretio Silicea* and *Inula racemosa* were extracted with water, concentrated them into extracts, and mixed them with the powders of six medicinal materials such as *Caryophylli Flos* and *Cinnamomi Cortex*, supplemented with starch and CM-NA, to prepare ESYWLRHW, and explored its therapeutic effect on NAFLD.

PPARs belong to the subfamily of the nuclear hormone receptor superfamily of transcription factors, including three subtypes of PPARα, PPARβ/δ and PPARγ (Monroy-Ramirez et al., 2021; Tanaka et al., 2017). After PPARs combines with retinoid X receptors to form heterodimers, its conformation changes and is activated, and then combines with peroxisome proliferator reaction elements, thereby initiating downstream gene transcription and participating in physiological processes such as lipid metabolism, cell proliferation and differentiation in the body (Bardot et al., 1993). Members of the PPARs family may alleviate the symptoms of NAFLD by regulating oxidative stress, inflammation, and IR (Peraldi et al., 1997; Luo et al., 2017). It is crucial to explore the changes of the PPAR signaling pathway during the treatment of NAFLD with ESYWLRHW.

We implemented a secondary development of the ESWWLRHW, resulting in the preparation of its concentrated pill form, ESYWLRHW. A systematic investigation into the material basis of ESYWLRHW was conducted. Subsequently, an integrated multi-omics approach—encompassing metabolomics, proteomics, and transcriptomics—was employed to preliminarily elucidate the therapeutic effects and underlying molecular mechanisms of ESYWLRHW against NAFLD. The overarching aim of this research is to clarify the pharmacologically active constituents, efficacy, and mechanistic pathways of ESYWLRHW, thereby providing a scientific foundation for the development of innovative therapeutic agents for NAFLD.

## 2 Materials and methods

### 2.1 Materials

All the medicinal materials used to prepare the ESYWLRHW were purchased from the Lotus Pond Chinese Medicinal Materials Wholesale Market. Enzyme-linked immunosorbent assay (ELISA) kits of Interleukin (IL)-13 (JYM0477Ra), IL-4 (JYM0647Ra), IL-1β (JYM0419Ra), IL-18 (JYM0650Ra), Catalase (CAT) (JYM0784Ra), SOD (JYM0267Ra) and GSH-PX (JYM0783Ra) were purchased from Wuhan jiyinmei biotech. HDL-C assay kit (A112-1-1), LDL-C assay kit (A113-1-1), T-CHO assay kit (A111-2-1), TG assay kit (A110-2-1), AST assay Kit (C010-1-1) and ALT assay Kit (C009-1-1) were obtained from Nanjing Jiancheng Bioengineering Institute. The high diet food (D12492) was purchased from SPF (Beijing) Biotechnology Co., Ltd. Obeticholic acid (I193491-5g), purchased from Shanghai Aladdin Biochemical Technology Co., Ltd. ESWWLRHW (20200501), purchased from Qinghai Qaidam High-Tech Pharmaceutical Co., Ltd. Enhanced BCA Protein Assay Kit (P0010), Primary Antibody Dilution Buffer (P0023A-100 mL), Trizol (R0016), purchased from Beyotime Biotechnology. NF-κB (T55034), purchased from Abmart Shanghai Co.,Ltd. NLRP3 (DF15549), Caspase1 (AF5418) and Nrf2 (AF0639),purchased from Affinity Biosciences. PPARα (66826-1-Ig), PPARγ (16643-1-AP), GAPDH (60004-1-Ig), purchased from Proteintech. SYBRPrime qPCR Set, purchased form Bioground.

### 2.2 HPLC-Q-TOF-MS/MS substance analysis of ESYWLRHW

HPLC-Q-TOF-MS/MS was used to study the components contained in ESYWLRHW.

Chromatographic parameters: The column was Agilent ZORBAX SB-C18 Rapid Resolution HD 2.1 × 100 mm 1.8 micron. Column temperature: 40 °C, mobile phase: 0.1% formic acid water (A), acetonitrile (B), binary linear gradient elution: 0–0.5 min, 2% B; 0.5–10 min, 2% B→95% B; 10–12 min, 95% B; 12–12.1 min, 95% B→2% B; 12.1–15 min, 2% B. Flow rate: 0.3 mL/min, injection volume: 2 μL.

Mass spectrum parameters: scanning time 0.662 s (primary) and 0.662 s (secondary); The collection time was 15 min. Collection range 50–1,200 Da; Atomizing gas flow rate 50 psi; Desolvent gas flow rate 50 psi; Air curtain gas flow rate 35 psi; Desolvent temperature 500 °C; Positive ion mode spray voltage 5500 V; Cluster removal voltage 80 V; Collision energy 10 V (first order), 50 V (second order).

Data processing method: MS-DIAL software was used to analyze the mass spectrometry data obtained by HPLC-QTOF-MS/ MS analysis by comparing the source-DIAL official website (http://prime.psc.riken.jp/compms/msdial/main.html) ESI MS (+) -MS/MS from the authentic standard database.

### 2.3 Animal model

After 1 week of adaptive feeding, 84 male SD rats (100–110 g) purchased from SPF Biotechnology Co., Ltd. (SYXK(Jing) 2024-0001) are divided into seven groups (*n* = 12) randomly, including Control group, Model group and Positive drug group (OCA, 25 mg/kg), ESYWLRHW-L (150 mg/kg), ESYWLRHW-M (300 mg/kg), ESYWLRHW-H (600 mg/kg) and ESWWLRHW (450 mg/kg). Control group was given the ordinary maintenance diet every day, and the other groups were fed with HFD for NAFLD modeling, and each group was given enough drinking water (Jiang et al., 2024). After 4 weeks of HFD feeding, one rat in Control group and one in Model group was randomly selected for dissection to detected whether the modeling was successful (Supplementary Figure S1). At the beginning of the fifth week, the Control group and Model group received 0.5% CMC-Na by intragastric administration, the rats in each administration group were received intervention drugs orally once daily for 6 weeks, and the Figure 2A is the animal experimental flow chart. During the experiment, the mental state and the changes of fur drinking and eating were observed, the weight and food intake were monitored weekly. Our animal experiments were approved by the Animal Ethics Committee of Sichuan University (K2024037) and were conducted according to ARRIVE guidelines.

After 6 weeks of drug intervention, the rats were fasted overnight with free access to water. Subsequently, the rats were anesthetized by intraperitoneal injection of 1% pentobarbital sodium and fixed in a supine position on a custom-made surgical platform. After disinfecting the chest and abdomen with 75% alcohol, a midline incision was made along the sternum from the xiphoid process to the neck to expose the thoracic cavity. Blood was then collected from the heart using vacuum blood collection tubes. The blood samples were allowed to stand at room temperature before being centrifuged at 4 °C and 3,500 rpm to separate the serum. The serum was carefully aliquoted into EP tubes and stored at −80 °C for subsequent analysis. Following blood collection, the liver and epididymal fat were rapidly dissected. After being rinsed with pre-cooled saline and blotted dry with sterile gauze, the tissues were accurately weighed. The left lobe of the liver was fixed in 4% paraformaldehyde overnight at 4 °C for subsequent histological processing and sectioning. The remaining liver tissue and epididymal fat were immediately snap-frozen in liquid nitrogen and then transferred to a −80 °C freezer for long-term storage. These collected biological samples (serum, fixed liver lobe, and frozen tissues) were used for downstream multi-omics and molecular analyses, including transcriptomics, proteomics, metabolomics, Western blot, qRT-PCR, and ELISA.

### 2.4 Histological and lipid evaluations

4% paraformaldehyde was used to fix the liver tissue with 24 h, then, the liver tissue was dehydrated, embedded in paraffin and cut into 5 μm sections. HE and ORO staining were performed according to the standard protocol. According to the study of Kleiner et al. (2005), NAFLD activity score (NAS) was performed on HE staining. ImageJ software was used to quantitatively analyze the positive area of oil red O staining.

### 2.5 Determination of liver function indexes

The activities of AST and ALT were measured by microplate method according to the kit instructions, substitute the OD value into the standard curve for calculation. Then, AST activity (Karman unit) × 0.48 × N is obtained by substituting AST activity (U/L) = into the standard curve. ALT activity (U/L) = the standard curve was substituted to obtain ALT activity (Karman unit) ×0.48 × N, and finally the serum AST and ALT activity were obtained. Where 0.482 is the conversion of Karman’s unit to U/L; N is the dilution ratio before sample test.

### 2.6 Determination of blood lipids

Serum levels of HDL-C, LDL-C, T-CHO and TG were measured according to kit instructions.

### 2.7 ELISA experiment of serum inflammatory factors

Standard, blank, and sample wells were sequentially set up on the pre-coated ELISA plate. All subsequent steps, including reagent addition, incubation, and washing, were performed strictly in accordance with the manufacturer’s instructions for the specific kit. After the final incubation and addition of the stop solution, the plate was immediately transferred to a microplate reader. The absorbance (Optical Density, OD value) of each well was measured at a primary wavelength of 450 nm. For each target inflammatory factor (IL-13, IL-4, IL-1β, and IL-18), the concentration in the serum samples from each experimental group was calculated by the standard curves.

### 2.8 ELISA experiment of oxidative stress factor in liver tissues

The liver tissue with a precise wet weight of about 50 mg was placed in EP tube, and PBS was added according to the weight to volume ratio of 1:10 tissue wet weight (mg) to PBS (μL), and 3-5 grinding steel balls were placed in the frozen grinding machine for grinding symmetrically. Centrifuge at 4 °C, 3,500 r/min for 15 min. The protein supernatant was determined by BCA test, and the contents of CAT, SOD and GSH-PX were determined accurately according to the ELISA kits, and corrected according to the total protein content of the sample.

### 2.9 Proteomic analysis

The frozen liver tissue was taken out, added with liquid nitrogen, fully ground, and an appropriate amount of ground product was transferred to high-speed centrifuge tube. An appropriate amount of lysis buffer was added, and protease and phosphatase inhibitors were supplemented to a final concentration of 1 mM. Sample was digested with trypsin and desalted, the LC-MS/MS was used to identify samples, and the specific parameters are shown in Supplementary Tables S1, S2. The mass spectrometry data were collected by DIA technology, and the spectrum matching, quantitative information extraction and subsequent statistical analysis were carried out. All mass spectrometry data were merged by PASER software, and the database retrieval of DIA mass spectrometry data and protein DIA quantitative analysis were completed. The search sequence file was uniprot-Rattus norvegicus-10116-2024.2.1.fasta. Pro DIA proteomics was performed by Shanghai Ouyi Biotechnology Co., Ltd. (Shanghai, China).

### 2.10 Transcriptome analysis

Total RNA was extracted from liver tissue samples according to the operating guidelines of TRIzol reagent. Subsequently, the NanoDrop 2000 spectrophotometer was used to detected the purity and concentration, and the Agilent 2100 Bioanalyzer system was used to assess RNA integrity. Subsequently, the sequencing library was constructed according to the VAHTS Universal V5 RNA-seq Library Prep kit operating manual. Finally, the library was sequenced using the Illumina Novaseq 6000 sequencing platform. Transcriptome sequencing of eukarya was performed by Shanghai Ouyi Biotechnology Co., Ltd. (Shanghai, China). Fastp v0.0.1, fastpc v0.11.9 and RseQC v4.0.0 software were used to control the quality of sequencing data. Hisat2 v2.1.0 software was used to compare the genomes. Using samtools v1.9 software to analyze bam files and sam files; gene quantification was performed using htseq-count v0.11.2 software. Biological repetition and paired difference analysis were performed using DESeq2 v1.22.2 software. Differentially expressed genes (DEGs) were screened according to the fold change | Fold Change | > 2 (i.e. | log2FC | > 1) and *q* < 0.05. Subsequently, GO function and KEGG Pathway enrichment analysis were performed on differentially expressed genes to further elucidate the biological functions of DEGs and the molecular mechanisms involved.

### 2.11 Metabolomic analysis

In this study, six rat liver tissue samples were randomly selected from each group of Control group, Model group and ESYWLRHW-H group for analysis. Level One 500 full-spectrum metabolomics was performed by Shanghai Ouyi Biotechnology Co., Ltd. (Shanghai, China). Sample pretreatment: 30 mg liver tissue samples were placed in a 1.5 mL centrifuge tube, added with grinding beads, 400 μL methanol-water, and transferred to the tissue grinding instrument, ground at a frequency of 60 Hz for 2 min. Then, ultrasonic extraction was carried out under the condition of ice water bath for 10 min, followed by standing at −40 °C for 2 h. The sample was centrifuged at 13,000 rpm and 4 °C for 20 min, and 150 μL of the supernatant was accurately sucked into the injection vial using a syringe for LC-MS analysis. The chromatographic parameters are shown in Supplementary Table S3, and the specific parameters of mass spectrometry are shown in Supplementary Table S4. The results were analyzed, *p*-value 1.0 was set, differential metabolites were screened, and pathway analysis was performed through the KEGG database.

### 2.12 RNA extraction and quantitativereal-time PCR (qRT-PCR)

Trizol was used to extract RNA from about 50 mg of liver tissue according to the operation steps. The density of RNA was detected by Nanodrop 2000 (Thermo Scientific). PrimeScriptrm RT reagent Kit gDNA Eraser (Takara, Japan) was used to remove genomic DNA and reverse transcription. SYBRPRIME PCR KIT (Fast HS) (Chongqing Baoguang Biotechnology Co., Ltd.) was used for qRT-PCR. The primers are shown in Supplementary Table S5.

### 2.13 Western blot

Cut about 50mg of liver tissue, add the RIPA lysis mixture, and then place it in a tissue grinder to prepare homogenization. The protein was separated by SDS-PAGE gel and transferred by PVDF membrane, with 5% skim milk blocked. The corresponding primary antibody and PVDF membrane were incubated overnight at 4 °C. The TBST was used to wash the membrane for three times, the secondary antibody was added and incubated for 1 h. After washing with TBST for 3 times, the enhanced chemiluminescence kit was used for exposure and photographing on the gel imager. The primary antibodies information: GAPDH (11f7886, Affinity Biosciences), PPARα (66826-1-Ig, Proteintech), PPARγ (AF6284, Affinity Biosciences), Nrf2 (AF0639, Affinity Biosciences), NLRP3 (DF7438, Affinity), PI3K (T40115, Abmart), NF-κB (TA5006M, Abmart), Caspase1 (AF5418, Affinity).

### 2.14 Statistical analysis

The experimental data were analyzed by GraphPad Prism 8.0, and presented as mean ± standard error of mean (± SEM). Normality and Lognormality Tests was used to evaluate the normality of the data. Data that followed a normal distribution were analyzed using One-way ANOVA followed by the Tukey *post hoc* test. Data that deviated from a normal distribution were analyzed with the Kruskal–Wallis *H* test, followed by Dunn’s *post hoc* test. The *p*-value <0.05 was considered to be significant.

## 3 Results

### 3.1 Substance analysis of ESYWLRHW

**FIGURE 1:**
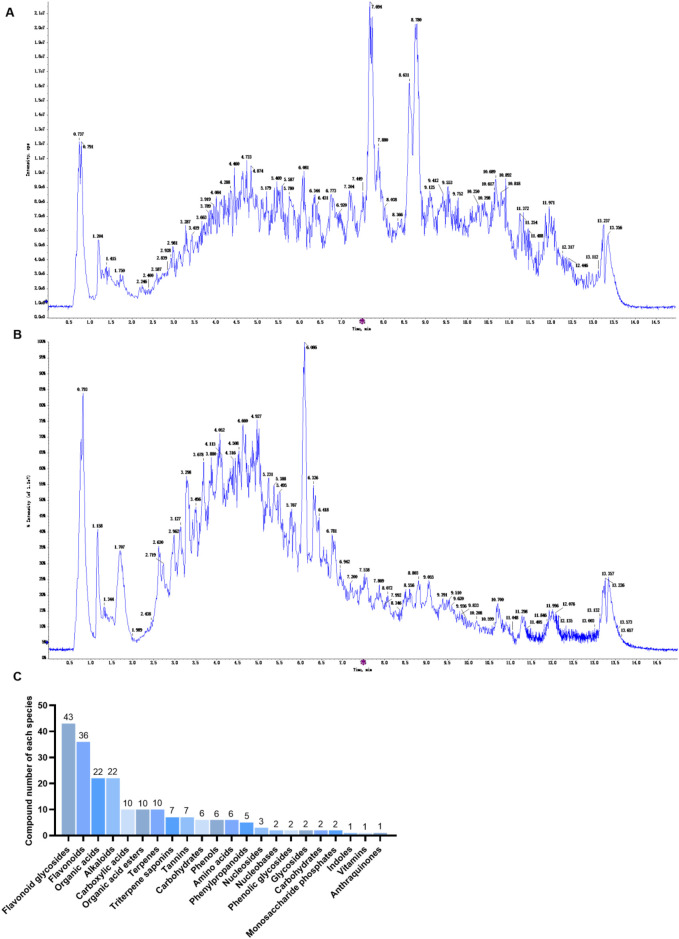
Substance analysis of ESYWLRHW. **(A)** Positive ion mode. **(B)** Negative ion mode. **(C)** Types of compounds in ESYWLRHW.

Positive and negative ion flow diagram of non-targeted analysis of ESYWLRHW is shown in Figures 1A,B, and total of 206 compounds were identified by high-resolution mass spectrometry quasi-molecular ion peak data combined with database analysis. The results of component identification are shown in Supplementary Table S6, and the identified compounds are classified (Figure 1C). ESYWLRHW contains 22 components. Each type of component from more to less in turn is flavonoid glycosides, flavonoids, organic acids, alkaloids, carboxylic acids, organic acid esters, terpenes, triterpenoid saponins, tannins, carbohydrates, phenols, amino acids, phenylpropanoids, nucleotides, nucleobases, phenolic glycosides, glycosides, carbohydrates, monosaccharide phosphates, indoles, vitamins, Anthraquinones.

### 3.2 ESYWLRHW alleviated the pathological condition of NAFLD rats

**FIGURE 2:**
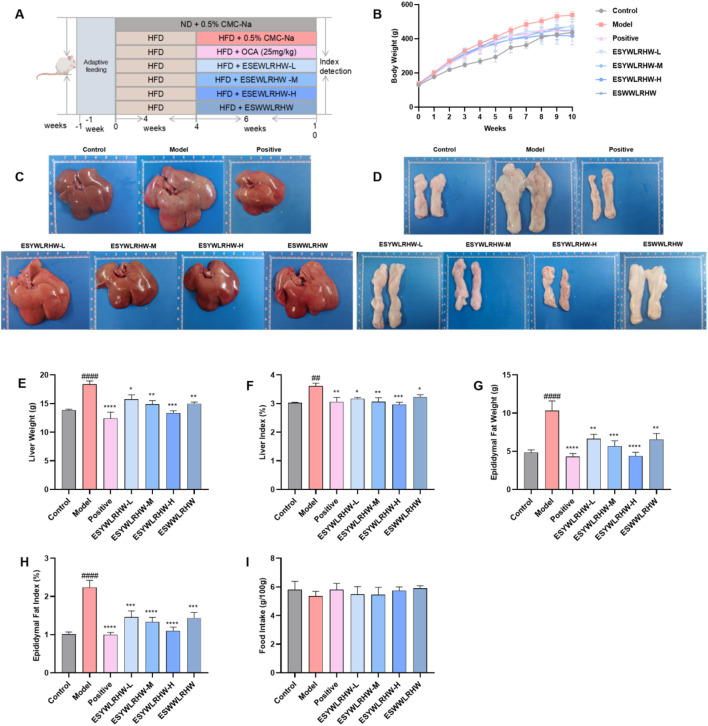
ESYWLRHW alleviated the pathological condition of NAFLD rats. **(A)** Animal experiment procedure. **(B)** The body weight. **(C)** Morphology of liver tissue. **(D)** Morphology of epididymal fat in each group. **(E)** Liver weight. **(F)** Liver index. **(G)** Epididymal fat weight in each group. **(H)** Epididymal fat index in each group. **(I)** Food intake in each group. *n* = 6. Compared to Control group: ^##^ *p* < 0.01, ^####^ *p* < 0.001; Compared to Model group: *p < 0.05, **p < 0.01, ***p < 0.005, ****p < 0.001.

Compared with the Control group, the weight gain trend of the rats fed with HFD was more obvious, and the fur was brighter and smoother. After drug intervention, each dose of ESYWLRHW, ESWWLRHW and OCA could inhibit the weight gain of NAFLD rats (Figure 2B). Compared with the model group, the liver morphology of rats in each administration group was significantly improved (Figure 2C). Liver weight (Figure 2E), liver coefficient (Figure 2F), epididymal fat weight (Figure 2G) and epididymal fat coefficient (Figure 2H) were also significantly improved by the treatment of ESYWLRHW. Among them, ESYWLRHW-H had the best effect after treatment, and the therapeutic effects of ESYWLRHW-M and ESYWLRHW-H were better than ESWWLRHW. At the same time, we monitored the food intake and found that there was no significant difference (Figure 2I). In addition, we also explored whether the doses of ESYWLRHW administration were toxic to the heart, spleen, lung and kidney of SD rats by HE staining. Under the 40-fold objective lens, there existed no difference of the important organs of each administration group as compared to Control group (Supplementary Figure S2), which indicates that each dose of ESYWLRHW has no obvious organ damage to rats.

### 3.3 ESYWLRHW improved liver morphology and fat deposition in NAFLD rats

**FIGURE 3:**
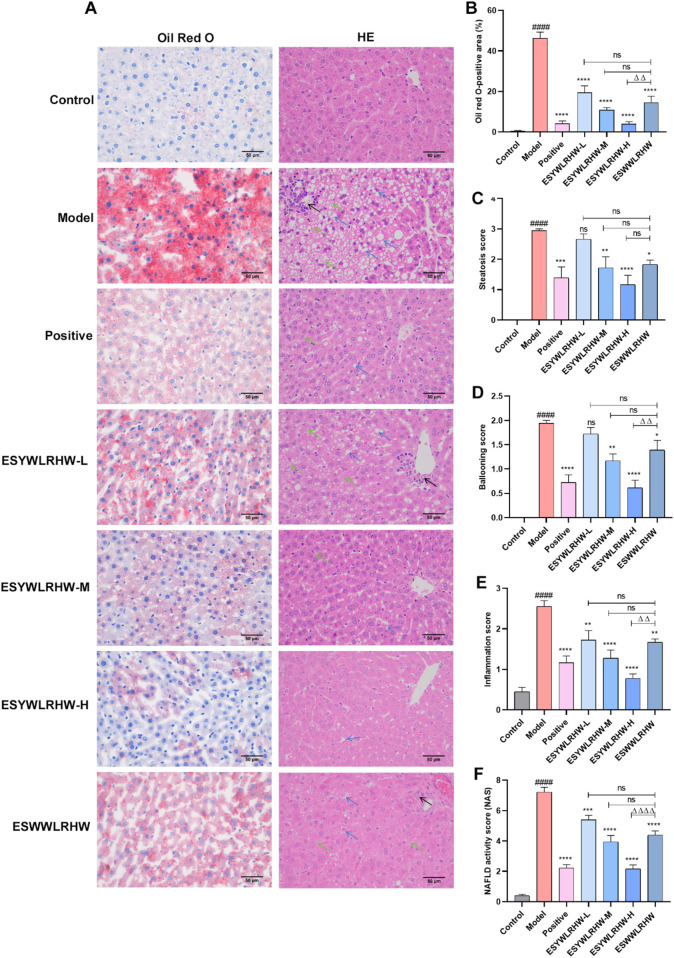
ESYWLRHW improved liver morphology and fat deposition in NAFLD rats. **(A)** HE and ORO staining. **(B)** ORO staining positive staining area. **(C)** Steatosis score. **(D)** Ballooning score. **(E)** Inflammation score. **(F)** NAFLD activity score. Black arrows represent lobular inflammation, blue arrows represent steatosis, and green arrows represent ballooning. *n* = 6. Compared to Control group: ^####^ *p* < 0.001; Compared to Model group: *p < 0.05, **p < 0.01, ***p < 0.005, ****p < 0.001; Compared to ESWWLRHW group: ^ΔΔ^ *p* < 0.01, ^ΔΔΔΔ^ *p* < 0.001.

Under the 40-fold objective microscope, liver tissue sections from the Control group exhibited a normal histological architecture with neatly arranged hepatocytes and no evident inflammatory cell infiltration (Figure 3A). In contrast, the Model group displayed pronounced pathological features of NAFLD, including marked hepatic steatosis characterized by numerous fat vacuoles and significant inflammatory cell infiltration Consequently, the NAS score and its individual components (steatosis, inflammation, and ballooning scores) were significantly elevated in the Model group compared to the Control group (Figures 3C–F). Treatment with ESYWLRHW notably ameliorated these histopathological alterations. The inflammatory infiltration, steatosis, and other relevant indices were significantly improved in all ESYWLRHW-treated groups compared to the Model group (Figures 3C–F). Notably, the medium- and high-dose ESYWLRHW groups (ESYWLRHW-M and ESYWLRHW-H) demonstrated superior therapeutic efficacy against NAFLD lesions compared to the ESWWLRHW group.

To specifically evaluate hepatic lipid deposition, we performed Oil Red O (ORO) staining, which selectively stains neutral lipids such as triglycerides. At 40× magnification, liver sections from the Control group showed minimal ORO-positive staining area and no observable steatosis or prominent lipid droplets (Figures 3A,B). Conversely, the Model group exhibited severe hepatic steatosis, as evidenced by a substantial increase in red-stained lipid droplets and a significantly expanded ORO-positive area compared to the Control group (Figures 3A,B). Pharmacological intervention in all treatment groups effectively reduced hepatic lipid accumulation. The number of lipid droplets and the ORO-positive area were markedly decreased, and hepatocyte morphology was considerably improved in the positive drug group, all ESYWLRHW administration groups, and the ESWWLRHW group relative to the Model group (Figures 3A,B). Consistent with the H&E findings, ESYWLRHW-M and ESYWLRHW-H exhibited a more potent effect in reducing liver fat deposition than ESWWLRHW.

### 3.4 ESYWLRHW improved liver function, dyslipidemia and inflammatory response in NAFLD rats

**FIGURE 4:**
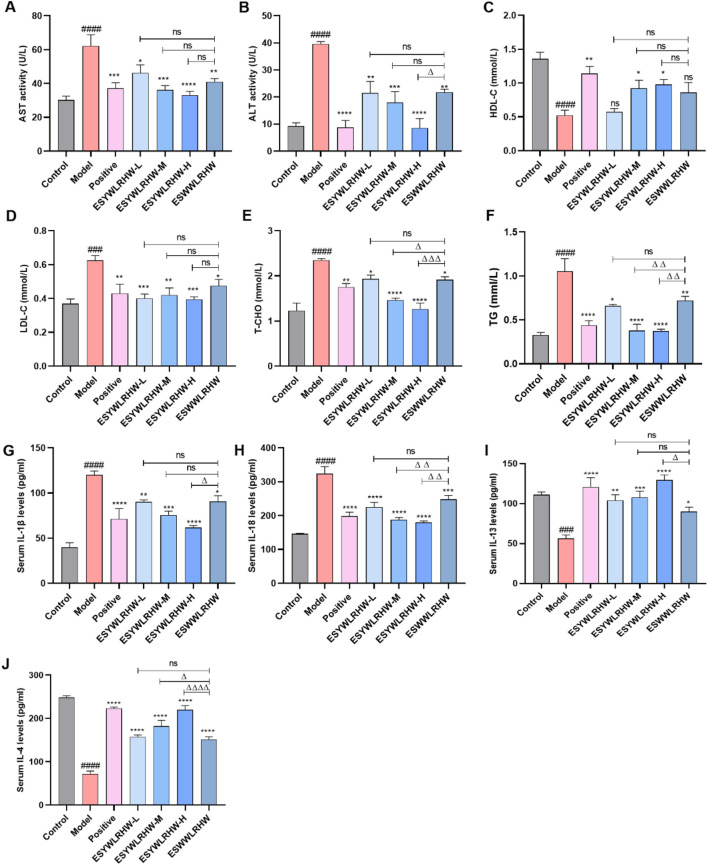
ESYWLRHW improved liver function, dyslipidemia and inflammatory response in NAFLD rats. **(A)** AST activity. **(B)** ALT activity. **(C)** HDL-C levels. **(D)** LDL-C levels. **(E)** T-CHO levels. **(F)** TG levels. **(G)** Serum IL-1β levels. **(H)** Serum IL-18 levels. **(I)** Serum IL-13 levels. **(J)** Serum IL-4 levels. *n* = 6. Compared to Control group: ^##^ *p* < 0.01, ^###^ *p* < 0.005, ^####^ *p* < 0.001; Compared to Model group: *p < 0.05, **p < 0.01, ***p < 0.005, ****p < 0.001; Compared to ESWWLRHW group: ^Δ^ *p* < 0.05, ^ΔΔ^ *p* < 0.01, ^ΔΔΔ^ *p* < 0.005, ^ΔΔΔΔ^ *p* < 0.001.

AST and ALT are crucial indicators in the evaluation of liver function, which may occur in a variety of diseases, and their increase means that liver function is impaired (Wu et al., 2023). AST and ALT activities in the Model group were significantly elevated compared to the Control group, indicating liver function impairment in the rats. Following treatment with ESYWLRHW, serum AST and ALT activities markedly decreased (Figures 4A,B). The development of NAFLD is closely associated with dysregulated lipid metabolism, with the most direct indicators being elevated levels of T-CHO, TG, and LDL-C, or reduced HDL-C levels. As shown in Figures 4C–F, the Model group exhibited significantly higher levels of LDL-C, TG, and T-CHO, along with a notable decrease in HDL-C, confirming the successful establishment of the NAFLD rat model. Treatment with varying doses of ESYWLRHW, positive controls, and ESWWLRHW resulted in a significant reversal of these trends (Figures 4C–F). Notably, the improvement in the four blood lipid parameters by ESYWLRHW-M and ESYWLRHW-H surpassed that of ESWWLRHW. Inflammation is another critical factor in NAFLD. Consequently, we assessed inflammatory markers and observed that serum levels of IL-13 and IL-4 in the Model group were significantly reduced, while levels of IL-18 and IL-1β were significantly elevated compared to the Control group (Figures 4G–J). This finding indicates a substantial inflammatory response in the Model group, and the administration of ESYWLRHW effectively reversed this response and ameliorated inflammation in NAFLD rats (Figures 4G–J).

### 3.5 ESYWLRHW improved arachidonic acid metabolism, primary bile acid biosynthesis and steroid hormone biosynthesis in NAFLD rats

**FIGURE 5:**
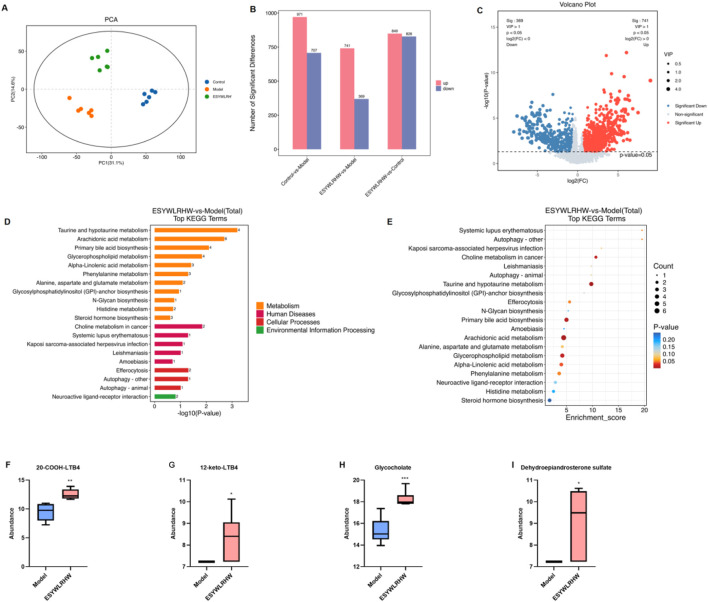
ESYWLRHW improved arachidonic acid metabolism, primary bile acid biosynthesis and steroid hormone biosynthesis in NAFLD rats. **(A)** PCA. **(B)** Number of significant differences. **(C)** Volcano diagram of differential metabolites. **(D)** KEGG enrichment analysis top20 histogram. **(E)** KEGG bubble diagram. **(F)** The abundance of 20-COOH-LTB4. **(G)** The abundance of 12-keto-LTB4. **(H)** The abundance of Glycocholate. **(I)** The abundance of Dehydroepiandrosterone sulfate. *n* = 6. Compared to Model group: *p < 0.05, **p < 0.01, ***p < 0.005.

We performed metabolomics analysis on the Control group, Model group, and ESYWLRHW group. Through principal component analysis (PCA) of metabolites in each group, it was found that the samples in each group converged into clusters, indicating that the differences within the group were small, and the samples in each group showed a significant aggregation trend (Figure 5A). The statistical figures of differential metabolites in each comparison group are shown in Figure 5B. The screening criteria were set as follows: *p*-value 1.0, and the volcano plot and cluster heat map analysis were drawn (Figure 5C). A total of 1,110 differential metabolites were screened in the ESYWLRHW group and the Model group, of which 741 were upregulated and 369 were downregulated. Through the KEGG database (https://www.genome.jp/kegg/), based on the KEGG identifier, the hypergeometric distribution test method was used to screen out the metabolic pathways that were significantly enriched in the differentially expressed metabolites. It was found that the three pathways of primary bile acid biosynthesis, arachidonic acid metabolism and steroid hormone biosynthesis were more obvious in the two comparison groups, and were selected as the metabolic pathways of EESYWLRHW in the treatment of NAFLD rats (Figures 5D,E). After the treatment of ESYWLRHW, the abundance of 12-keto-LTB4, 20-COOH-LTB4, GCA and DHEAS in NAFLD rats was significantly increased as compared to Model group (Figures 5F–J).

### 3.6 ESYWLRHW improved NAFLD symptoms in rats through PPARs

**FIGURE 6:**
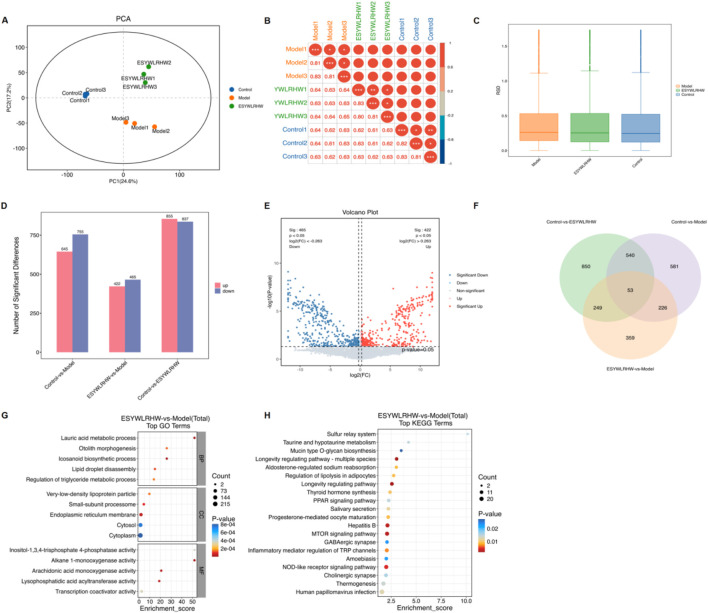
Proteomics analysis to explore the mechanism of ESYWLRHW in the treatment of NAFLD. **(A)** PCA. **(B)** Sample correlation diagram. **(C)** RSD distribution box plot. **(D)** Statistical chart of differential proteins. **(E)** Volcano diagram of differential proteins. **(F)** Venn diagram. **(G)** GO enrichment analysis top15 Bubble diagram. **(H)** KEGG enrichment analysis top20 bubble diagram.

We performed proteomics analysis on the group of Control, Model and ESYWLRHW. As shown in Figure 6A, samples in each group were clustered into bundles, and samples between groups were discrete, indicating that the similarity within each group was high. From the sample correlation analysis diagram, it can be seen that the repeatability of the samples every group is good, and the difference among the groups is large (Figure 6B). The relative standard deviation RSD was used for the results obtained, and the RSD distribution box line diagram (Figure 6C) was drawn to test whether the quantitative results of the samples were statistically consistent. We found that the repeatability of each group of samples was fine and could be used for subsequent analysis. We counted the differential proteins in each comparison group, and plotted histograms (Figure 6D), volcanoes (Figure 6E), and Venn diagrams (Figure 6F). Compared with the ESYWLRH group and the Model group, a total of 887 differential proteins were identified, including 422 upregulated proteins and 465 downregulated proteins. Then, the KEGG pathway enrichment and GO functional enrichment analysis were performed on the screened differential proteins in the ESYWLRHW and Model group, and plotted bubble plots (Figures 6G,H). It was found that ESYWLRHW may improve NAFLD through Longevity regulating pathway, MTOR signaling pathway, PPAR signaling pathway and so on.

**FIGURE 7:**
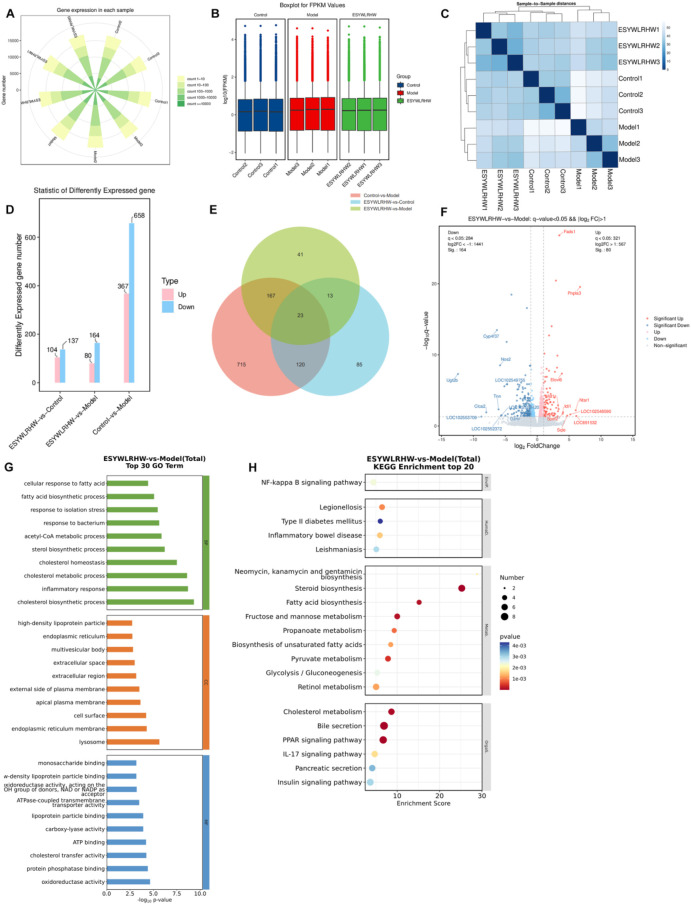
Transcriptomics analysis to explore the mechanism of ESYWLRHW in the treatment of NAFLD. **(A)** Statistics of the number of detected genes. **(B)** Box line diagram. **(C)** Sample-to-Sample cluster analysis results. **(D)** Statistical histogram of differentially expressed genes. **(E)** Venn diagram. **(F)** Volcano diagram of DEGs. **(G)** Differential gene GO enrichment analysis histogram. **(H)** KEGG enrichment analysis TOP 20 bubble diagram.

Transcriptome analysis was performed on the group of Control, Model and ESYWLRHW. We counted the detected genes, and drawing the statistical map of the number of detected genes (Figure 7A), and the FPKM standardization method was used to explore the intra-group differences and inter-group differences of each tissue sample. The FPKM value was used to correct the sequencing depth and gene length in the original sequencing results of the sample, and the box plot (Figure 7B) was drawn. It was found that the gene dispersion of each group of samples was low, and the dispersion of gene expression value distribution between each sample was basically the same. Differentially expressed genes (DEGs) between groups can be analyzed. Then through the cluster analysis of the gene expression of each group of samples, as shown in Figure 7C, the color of the samples in each group was darker and the distance was closer, suggesting that the samples in experiment had better repeatability. Next, we screened differential genes and drew the histogram (Figure 7D), Venn diagram (Figure 7E) and volcano diagram (Figure 7F). A total of 244 DEGs were screened, of which 80 were upregulated and 164 were downregulated between the Model and ESYWLRHW group. We performed GO functional enrichment analysis (Figure 7G) and KEGG pathway enrichment analysis (Figure 7H) on the DEGs of the ESYWLRHW group and the Model group screened above, and found that ESYWLRHW may improve NAFLD through NF-kappa B signaling pathway, Steroid biosynthesis PPAR signaling pathway and so on.

**FIGURE 8:**
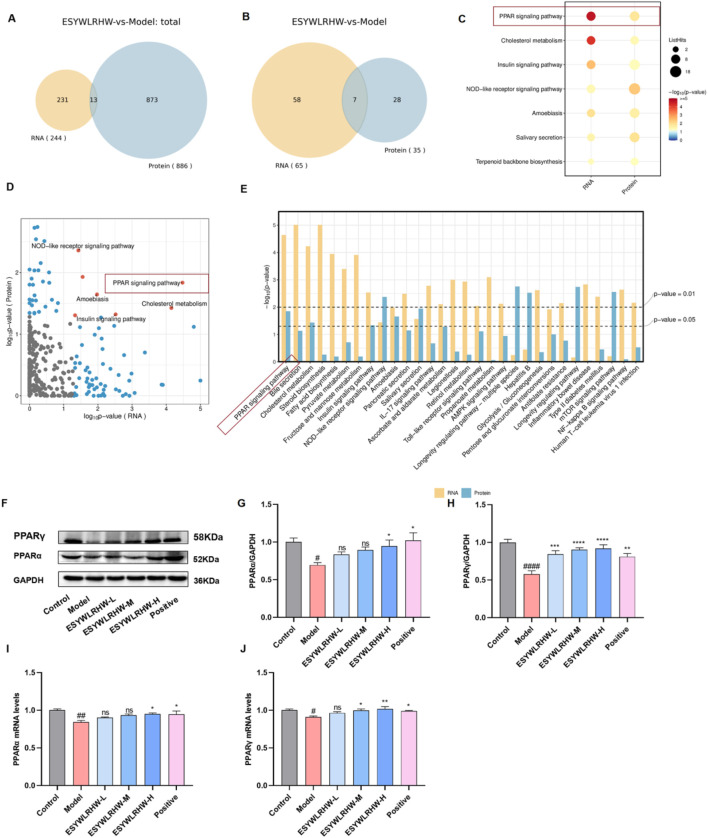
Joint analysis and verification of proteomics and transcriptomics. **(A)** Intersection analysis diagram of DEGs. **(B)** Intersection analysis diagram of KEGG pathways. **(C)** KEGG pathway bubble diagram. **(D)** KEGG pathway scatter plot. **(E)** KEGG pathway histogram. **(F)** Western blot bands of PPARα, PPARγ and GAPDH proteins. **(G)** Expression of PPARα protein. **(H)** Expression of PPARγ protein. **(I)** PPARα mRNA levels. **(J)** PPARγ mRNA levels. The data are shown as the mean ± SEM (*n* = 3). Compared to Control group: ^#^ *p* < 0.05, ^##^ *p* < 0.01, ^####^ *p* < 0.001; Compared to Model group: *p < 0.05, **p < 0.01, ***p < 0.005, ****p < 0.001.

We analyzed the differential genes in proteomics and transcriptomics reports, and plotted the Venn diagram (Figure 8A). Then, the results of KEGG Total enrichment analysis were analyzed jointly, and the significantly (*p*-value < 0.05) enriched pathways in each group were selected for intersection analysis. Extract the simultaneously significantly enriched KEGG pathways and draw a Venn diagram (Figure 8B). The results showed that a total of seven pathways were significantly enriched in both omics. The bubble diagram (Figure 8C), scatter diagram (Figure 8D) and histogram (Figure 8E) of the-log10 (*p*-value) of the pathway in the two omics were drawn to further show the significance of the pathway in the two groups. The results showed that the PPAR signaling pathway was more significant in proteomics and transcriptomics. Therefore, we verified the regulation of ESYWLRH on PPARs family proteins in NAFLD rats by WB and qRT-PCR experiments. Compared with the Control group, the mRNA and protein expression levels of PPARα and PPARγ in the Model group were significantly decreased. After 6 weeks of ESYWLRHW intervention, the protein and mRNA expression levels of PPARα and PPARγ were increased in a dose-dependent manner.

### 3.7 ESYWLRHW improved oxidative stress and inflammation in NAFLD rats by regulating PPARs and its downstreams

**FIGURE 9:**
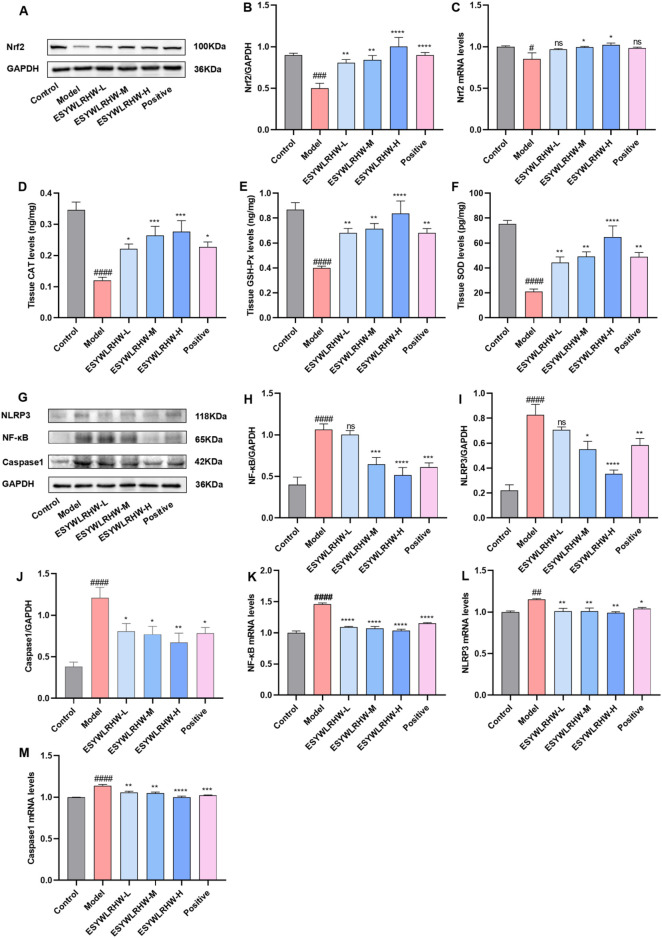
ESYWLRHW can improve oxidative stress, inflammation and insulin resistance in NAFLD rats. **(A)** Western blot bands of Nrf2 and GAPDH proteins. **(B)** Expression of Nrf2 protein. **(C)** Nrf2 mRNA levels. **(D)** Tissue CAT levels. **(E)** Tissue GSH-Px levels. **(F)** Tissue SOD levels. **(G)** Western blot bands of NLRP3, NF-κB, Caspase1 and GAPDH proteins. **(H–J)** NF-κB, NLRP3 and Caspase1 levels. **(K–M)** NF-κB, NLRP3, Caspase1 mRNA levels. Compared to Control group: ^##^ *p* < 0.01, ^###^ *p* < 0.005, ^####^ *p* < 0.001; Compared to Model group: *p < 0.05, **p < 0.01, ***p < 0.005, ****p < 0.001.

PPARγ can directly bind to the PPRE on the Nrf2 promoter to promote the expression of Nrf2, which in turn affects the expression of oxidative stress-related factors such as CAT, GSH-PX and SOD downstream of Nrf2 (Chen et al., 2023). Therefore, we detected the expression of the above Nrf2 pathway and its downstream cytokines. As shown in Figures 9A–F, the Nrf2 protein and mRNA levels in Model group were significantly decreased as compared to Control group. After ESYWLRHW administration, Nrf2 protein and mRNA levels were increased to varying degrees, showing a certain dose dependence. Compared with the Control group, the expression of antioxidant enzymes CAT, GSH-PX and SOD downstream of Nrf2 in the Model group was significantly decreased, while ESYWLRHW could significantly upregulate the levels of CAT, GSH-PX and SOD.

A number of studies have shown that activation of PPARα and PPARγ can affect the NF-κB signaling pathway, thereby inhibiting the production of inflammatory factors, and exerting anti-inflammatory effects (Delerive et al., 1999; Chung et al., 2000). Therefore, in this study, we used WB and qRT-PCR to determine the protein and mRNA expression of PPARγ/NF-κB/NLRP3/Caspase1 signaling pathway,and the experimental results are shown in Figures 9G–M. The levels of NF-κB, NLRP3 and Caspase 1 protein and mRNA in the Model group were significantly increased as compared to Control group. The levels of pro-inflammatory factors IL-18 and IL-1β in the serum of the Model group were significantly increased (Figures 3G,H). After treatment with different doses of ESYWLRHW, the expression levels of NF-κB, NLRP3 and Caspase1 protein and mRNA, as well as the expression levels of pro-inflammatory factors IL-18 and IL-1β in serum of rats in each administration group were decreased as compared to Model group. Among them, ESYWLRHW-M and ESYWLRHW-H groups could significantly inhibit the expression of inflammation-related proteins, genes and cytokines.

## 4 Discussion

NAFLD is a clinicopathological syndrome caused by excluding alcohol and other liver-damaging factors, and its pathological changes are mainly concentrated in the lobular area of the liver. The characteristic of this disease is the appearance of vesicular lipid deposition and excessive fat accumulation in liver cells (Liu and Wang, 2024). NAFLD includes a variety of pathological states ranging from simple steatosis to inflammatory lesions. Among them, the most common clinical types include non-alcoholic simple steatosis and nonalcoholic steatohepatitis (NASH) and so on (Guo et al., 2022; Friedman et al., 2018). At present, there is a lack of drugs for the treatment of NAFLD in clinical practice, and traditional Chinese medicine compound has achieved certain curative effect in the treatment of NAFLD, such as Qige Decoction (Fan et al., 2025), Linghe granules (Hu et al., 2025) and so on, we turned our attention to traditional Chinese and Tibetan combination preparations. ESWWLRHW is a proven prescription of the Xizang people.which is a compound preparation composed of Xizang medicinal materials such as *Meconopsis quintuplinervia* Regel, *Bambusae Concretio Silicea* and *Inula racemosa*, and is used for liver-related diseases such as liver and stomach congestion and pain, liver cirrhosis and hepatomegaly (Chen et al., 2009; Basang and Ciren, 2018). ESYWLRHW is a concentrated pill improved on the basis of ESWWLRHW. There exists great significance to explore whether it has therapeutic effects on NAFLD, the superiority or inferiority of its effects compared with the original drug ESWWLRHW, and the potential mechanism by which it improves NAFLD. In this study, we demonstrated that ESYWLRHW can improve the weight gain trend, liver morphological changes and epididymal fat weight of NAFLD rats, and improve abnormal indicators such as inflammation, liver fat deposition, liver function and lipid disorders in NAFLD rats. Moreover, the improvement effects of medium and high doses of ESYWLRHW on the above indicators were better than those of the original drug ESWWLRHW, and each dose of ESYWLRHW did not have a significant effect on the important organs of rats.

NAFLD is a typical metabolic disease (Eslam et al., 2020). Therefore, we studied the effect of ESYWLRHW on the metabolic status of NAFLD rats by metabolomics. The results indicated that significant changes occurred in 12-keto-LTB4, 20-COOH-LTB4, Glycocholate and DHEAS in NAFLD rats after administration of ESYWLRHW, which were respectively involved in biosynthesis of steroid hormone and primary bile acid biosynthesis, and arachidonic acid metabolism.

Arachidonic acid is a polyunsaturated fatty acid mainly present in cell membranes and can be converted into metabolites that trigger inflammatory responses through multiple pathways (Wu et al., 2024; Li J. et al., 2025). Leukotriene B4 (LTB4) is a bioactive metabolite produced by arachidonic acid through a 5-lipoxygenase coupling reaction. It undergoes cytochrome p-450-dependent ω-hydroxylation and then secondary oxidation to ω-hydroxylation to inactivate it. Finally, 20-COOH-LTB4 was generated (Wheelan et al., 1994). It binds to the BLT1 receptor with high affinity, inhibits the LTB4-mediated neutrophil response, migration, granulation and leukotriene biosynthesis. 12-keto-LTB4 is another product after the metabolism of LTB4, and its efficacy in stimulating neutrophils is greatly reduced (Powell et al., 1996). Another study demonstrated (Primiano et al., 1998) that dithiothione inducible gene 1 (DIG-1) can catalyze the dehydrogenation of LTB4 to 12-keto-LTB4, inhibiting the pro-inflammatory effect of LTB4. In our findings, ESYWLRHW might change the levels of 12-keto-LTB4 and 20-COOH-LTB4 in NAFLD rats through the arachidonic acid metabolic pathway, thereby improving the inflammation in NAFLD rats.

Bile acids synthesis is mainly occurred in the liver. Cholesterol is converted into free primary bile acids in the liver, including cholic acid and chenodeoxycholic acid, under the combined action of various enzymes. Then, under the action of other enzymes in the body, it is further metabolized and combines with glycine or taurine to form conjugated primary bile acids, including Glycocholate (GCA) (Shen et al., 2024). Studies have found that glycine cholic acid can significantly enhance lipase activity, accelerate lipid catabolic metabolism, and simultaneously stimulate bile secretion (Li P. et al., 2025). It can also suppress the content of LTB4 of rats (Li, 2006) and the NO in the serum of arthritis rats (Guan et al., 2009), which has a certain anti-inflammatory effect. GCA can increase the activities of GSH-PX and SOD to exert antioxidant capacity (Wu L. et al., 2020). In our research, ESYWLRHW increased the expression of GCA in NAFLD rats by regulating the primary bile acid metabolism pathway, exerting anti-inflammatory and antioxidant effects.

DHEAS, a biologically active hormone from the adrenal gland, is a metabolite of Dehydroepiandrosterone (DHEA) (Allolio and Arlt, 2002). Researchers have demonstrated that DHEAS can prevent coronary artery and aortic sclerosis, inhibit vascular proliferation and reducing blood lipids (Lin et al., 2025). DHEAS could stimulate lipolysis of adipose tissue and block adipogenesis *in vitro* (Stelfa et al., 2025). It also has beneficial effects on a variety of pathophysiological problems. Which was regarded as a longevity hormone and plays a crucial role in preventing cancer (Ratko et al., 2025), protecting the cardiovascular system, and improving obesity and insulin resistance (Lasco et al., 2001; Allolio and Arlt, 2002). Low serum levels of DHEAS are associated with pathological conditions such as prostate (Stahl et al., 1992) and rheumatoid arthritis (Deighton et al., 1992), especially with certain characteristics of obesity, such as high body mass index and obesity (Pergola et al., 1991; Tchernof et al., 1995) or central fat accumulation (Pergola et al., 1991). In this study, ESYWLRHW could increase DHEAS content in the liver of NAFLD rats, suggesting that ESYWLRHW may improve the symptoms of NAFLD through the pathway of steroidal biotin synthesis.

PPARs are a type of nuclear hormone receptor protein (Xi et al., 2020; Mirza et al., 2019), which have been considered as a key regulatory factor involved in metabolic diseases and play a significant part in metabolism of glucose and lipid (Li et al., 2024). Based on the combined results of proteomics and transcriptomics in this study, it was found that ESYWLRHW may play a role through the PPAR signaling pathway, and the WB and qRT-PCR were used to detected the key proteins of this pathway: PPARα and PPARγ. It was found that ESYWLRHW treatment could increase the protein and mRNA expression levels of PPARα and PPARγ in NAFLD rats significantly. Subsequently, we verified the signaling pathways associated with the PPARs family proteins to clarify how ESYWLRHW improves the symptoms of NAFLD through PPARs.

According to the “multiple-hit” theory, oxidative stress is a key driver in the pathogenesis and progression of NAFLD (Serviddio et al., 2013). Nrf2, a crucial transcription factor (Baird and Yamamoto, 2020), plays a central regulatory role in cellular defense mechanisms by protecting cells from oxidative stress and inflammatory damage. It exerts potent antioxidant effects primarily through the transcriptional activation of genes encoding various antioxidant enzymes, such as SOD and GSH-Px. These enzymes are essential for scavenging harmful free radicals, which can mitigate oxidative damage to cellular structures and functions (Kang et al., 2025). PPARγ activation has been shown to upregulate Nrf2 expression (Cho et al., 2010), whereas PPARγ deficiency reduces Nrf2 levels in mouse lung tissue (Collins et al., 2009). PPARγ can directly bind to PPRE on the Nrf2 promoter to promote the expression of Nrf2, thereby improving the oxidative stress of the body. In our study, treatment with ESYWLRHW upregulated PPARγ levels in NAFLD rats. This upregulation further elevated the expression of Nrf2 and subsequently promoted the expression of downstream antioxidant enzymes, including CAT, GSH-Px, and SOD, collectively contributing to the hepatoprotective effects.

In the context of NAFLD, persistent inflammatory responses occur within the liver (Paternostro and Trauner, 2022). NF-κB is a key transcription factor and plays a core role in the occurrence and development of inflammation (Lawrence, 2009). Upon activation, NF-κB promotes the transcription of genes encoding NLRP3, pro-IL-1β, and pro-IL-18. NLRP3 further activates caspase-1, enhances macrophage activity, and regulates inflammatory responses (Shan et al., 2008). Under physiological conditions, NF-κB remains inactive through binding to its inhibitory protein IκBα, while PPARα can directly interact with NF-κB subunits, compete for transcriptional activation sites, and thereby suppress the transcription of pro-inflammatory genes, ultimately inhibiting the inflammatory response (Delerive et al., 1999). Once activated, PPARγ interacts with inflammatory factor transcription factors such as NF-κB, STAT, and AP-1 (Dubois et al., 2017), participates in inflammatory signal transduction, and can reduce the expression of pro-inflammatory cytokines. Accordingly, we detected the related proteins of the NF-κB pathway and found that ESYWLRHW can inhibit the expression of NF-κB, NLRP3 and Caspase1 proteins by up-regulating the expression levels of PPARα and PPAR, and reducing the expression levels of pro-inflammatory factors IL-18 and IL-1β in NAFLD rats, and alleviate the inflammatory response.In summary, our findings indicates that ESYWLRHW can alleviate the symptoms of NAFLD by regulating three metabolic pathways: arachidonic acid metabolism, biosynthesis of primary bile acids, and biosynthesis of steroid hormones. It can also affect downstream oxidative stress and inflammation by regulating the expression of PPARα and PPARγ (Figures 8F–J), ultimately alleviating the symptoms of NAFLD. It is worth noting that after conducting a statistical analysis of the natural agonists of PPARγ, it was found that the majority are flavonoids or isoflavonoids (Wu Y. et al., 2020). Meanwhile, in our material analysis of ESYWLRHW, it was found that 43 flavonoid glycosides and 36 flavonoids in ESYWLRHW (Figure 1C). In the future, we will explore the pharmacodynamically active substances of ESYWLRHW through blood component analysis, and further explored the specific mechanism of the interaction between these compounds and PPARs through *in vitro* and *in vivo* experiments, thereby laying a foundation for the further development and utilization of ESYWLRHW.

## Data Availability

The transcriptomics and metabolomics data presented in the study are deposited in the China National Center for Bioinformation repository. The transcriptomics data (accession number: CRA036466) can be found here: https://ngdc.cncb.ac.cn/gsa/browse/CRA036466. The metabolomics data (accession number: OMIX014164) can be found here: https://ngdc.cncb.ac.cn/omix/release/OMIX014164. The proteomics data have been deposited to the iProX repository. And the data (accession number: IPX0015044000) can be found here: https://www.iprox.cn/page/project.html?id=IPX0015044000.
