# Supplementary material for: Study on the effect and mechanism of Ershiyiwei Lvronghao concentrated Pills in the treatment of nonalcoholic fatty liver disease
Source: Front Pharmacol. 2026 Jan 22;16:1725610. doi: 10.3389/fphar.2025.1725610 (PMC12872746; doi:10.3389/fphar.2025.1725610)
Supplement: Supplementary file 1 [file Supplementaryfile1.docx]

Supplementary Material

# Methods

## Preparation of ESYWLRHW

*Meconopsis quintuplinervia* Regel 100g, *Bambusae Concretio Silicea* (the dried mass of the secretions of *Bambusa textilis* McClure) 50g, *Inula racemosa* (the dry roots of *Inula racemosa* Hook. f.) 50g, *Vitis vinifera* L. 30g, *Milnep-edwards* 40g, *Carthami Flos* (the dried flowers of *Carthamus tinctorius* L.) 70g, *Herba veronicae* 70g, *Fructus Sinopodophylli* (*Saxifraga umbellulata* Hook. f. & Thomson) 80g, *Semen Herpetospermi* (the dried mature seeds of *Herpetospermum caudigerum* Wall. ex Chakrav.) 30g, *Piperis Longi Fructus* (the dried mature clusters of *Piper longum* L.) 20g, *Phyllanthi Fructus* (the dried mature fruits of *Phyllanthus emblica* L.) 100g, *Zingiberis Rhizoma* (the dried rhizome of *Zingiber officinale* Rosc.) 30g, *Glycyrrhizae Radix Et Rhizoma* (the dried rhizome of *Glycyrrhiza uralensis* Fisch.) 50g, *Herba Dracocephali Tangutici* (*Dracocephalum tanguticum* Maxim.) 80g and *Chebulae Fructus* (the dried mature fruits of *Terminalia chebula* Retz.) 100g. A total of 15 medicinal herbs were crushed and passed through No.2 sieve. 12 times the amount of water was added. After heating and reflux extraction for 1 h, the residue was filtered through a 420-mesh polypropylene sieve. The residue was recirculated again, and this process was repeated three times. The filtrates were combined and concentrated at 60 ℃ to a 1.20-density extract for later use. Grind 30 g of *Caryophylli Flos* (the dried flower buds of *Eugenia caryophyllata* Thunb.), 30 g of *Cinnamomi Cortex* (the dried bark of *Cinnamomum cassia* (L.) J.Presl), 40 g of *Aquilariae Lignum Resinatum* (Aquilaria sinensis ( Lour.) Gilg), 10 g of *Croci Stigma* (the dried stigmata of *Crocus sativus* L.), 70 g of Calcitum and 50 g of *Aucklandiae Radix* respectively, and pass through a 100-mesh pharmacopoeia sieve for later use. After mixing the extract and the medicinal powder, add the excipients. Excipients: medicinal powder = excipients (starch: CMC-Na) : medicinal powder (0.65:0.35) : 1 to make pills. The pills were dried in an electric heating blower drying oven at 60 ℃ for 14 h, and the ESYWLRHW were obtained.

## Dose conversion of ESWWLRHW and ESYWLRHW

ESWWLRHW is a traditional Xizang medicine compound preparation (pill). The daily dosage of adults is 5 g/d. According to the equivalent dose conversion formula of animal experiment, the dose of rats is Y mg/kg = human dose X mg/kg × 70 kg × 0.0018 ÷ 0.2kg = 6.3X mg/kg, and the dose of rats is 450mg/kg/d. In the process of preparing ESYWLRHW, taking 0.1 times the prescription amount of medicinal materials as an example, we finally obtained ESYWLRHW (g) : ESWWLRHW (g) ≈ 1 : 1.5. Therefore, the medium dose of ESYWLRHW we selected was 300mg/kg/d, which corresponds to the 450 mg/kg/d dose of ESWWLRHW. On this basis, we adjusted the dosage up and down to obtain the low dose of 150mg/kg/d and the high dose of 600mg/kg/d of ESYWLRHW.

# Supplementary Figures and Tables

## Supplementary Figures

##
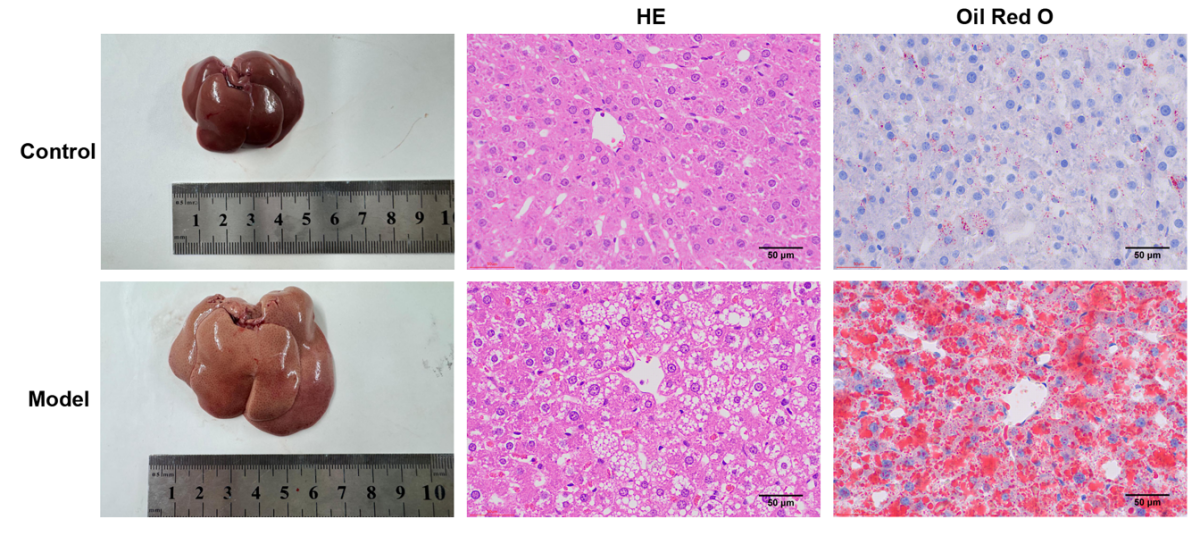


## Supplementary Figure 1. Liver morphology of rats after four weeks of modeling. (The scale bar is 50 μm).


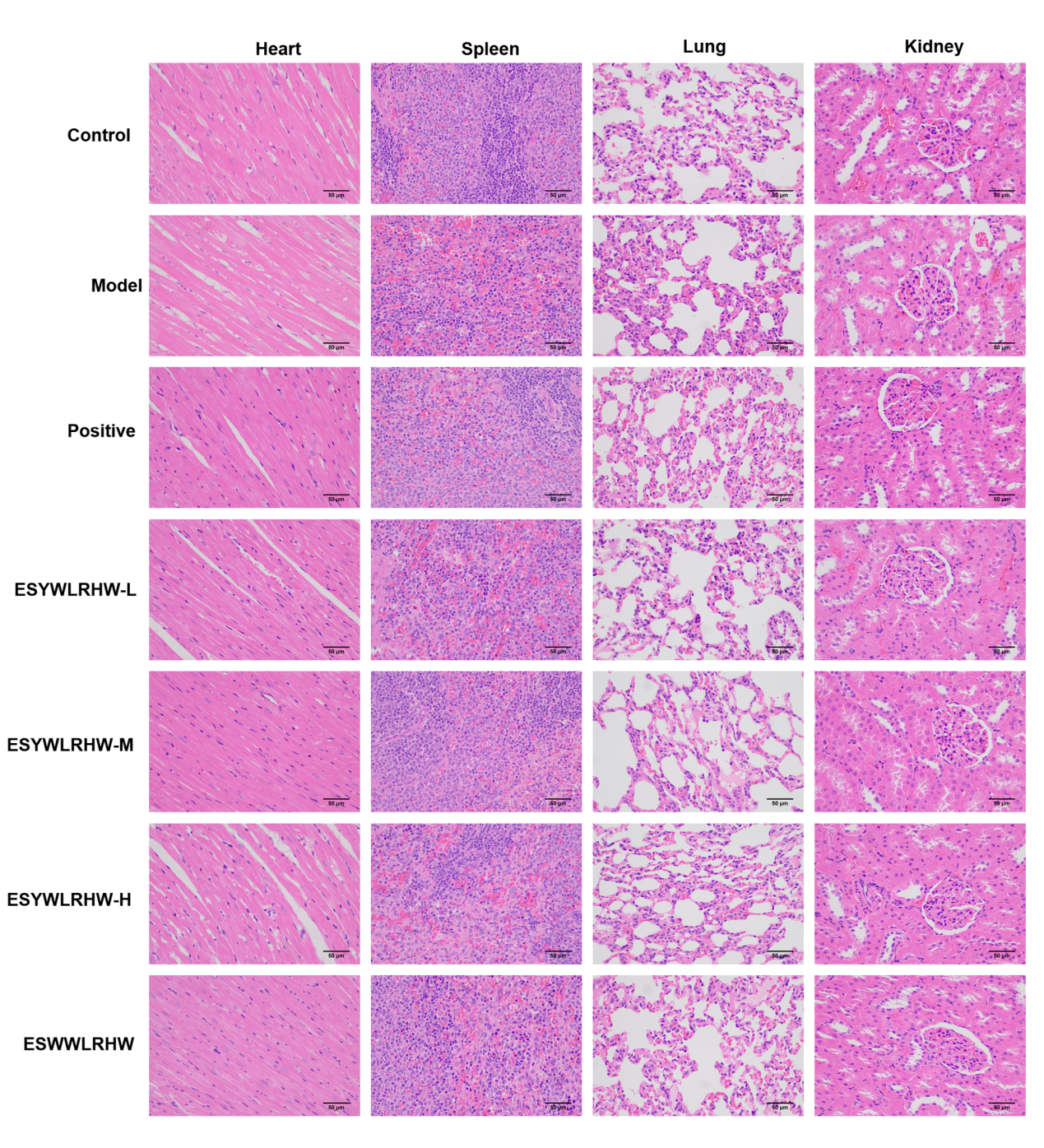


**Supplementary Figure 2.** HE staining results of heart, spleen, lung and kidney of rats in each group. (The scale bar is 50 μm).

## Supplementary Tables

Table. S1. Gradient elution

| Time（min） | Gradient | Flow rate（μL/min） |
| --- | --- | --- |
| 0 | 6% B | 0.7 |
| 7.5 | 28% B | 0.4 |
| 9 | 80% B | 0.7 |
| 10 | 80% B | 0.7 |

Table. S2. DIA mass spectrometry conditions

| Items | Para. |
| --- | --- |
| Capillary | 1.6KV |
| Dry Temperature | 180℃ |
| Dry Gas | 3.2 L/min |
| Mass Range | 300-1500 m/z |
| Ion Mobility | 0.7-1.3 |

Table. S3. Elution gradient

| Time (min) | A% | B% |
| --- | --- | --- |
| 0 | 95 | 5 |
| 2 | 95 | 5 |
| 4 | 70 | 30 |
| 8 | 50 | 50 |
| 10 | 20 | 80 |
| 14 | 0 | 100 |
| 15 | 0 | 100 |
| 15.1 | 95 | 5 |
| 16 | 95 | 5 |

Table. S4. Mass spectrometry parameters

| Parameters | Positive ions | Negative ions |
| --- | --- | --- |
| Spray Voltage （V） | 3800 | -3200 |
| 2Capillary Temperature （°C） | 320 | 320 |
| Aux gas heater temperature （℃） | 350 | 350 |
| Sheath Gas Flow Rate （Arb） | 35 | 35 |
| Aux gas flow rate （Arb） | 8 | 8 |
| S-lens RF level | 50 | 50 |
| Mass range （m/z） | 70-1050 | 70-1050 |
| Full ms resolution | 60000 | 60000 |
| MS/MS resolution | 15000 | 15000 |
| NCE/stepped NCE | 10，20，40 | 10，20，40 |

Table. S5. Primer information

| Gene | Forward Primer（5->3） | Reverse Primer（5->3） |
| --- | --- | --- |
| PPARα | CGATGCTGTCCTCCTTGATGAAC | GATGTCGCAGAATGGCTTCCTC |
| PPARγ | GAACGTGAAGCCCATCGAGGAC | GGAGCACCTTGGCGAACAGC |
| Nrf2 | TGCCTTCCTCTGCTGCCATTAG | CCGTGCCTTCAGTGTGCTTC |
| NF-κB | CGGTTACGGGAGATGTGAAGATG | GAAGGTGGATGATGGCTAAGTGTAG |
| NLRP3 | CCTGGTCTGCTGGATTGTGTGC | AGTCGTGGTCTTGGAGGTCTGG |
| Caspase 1 | CTACAGATGCCAACCACTGAAAGG | TCCCAACACAGGTACATAAGAATGAAG |

**Table. S6.** Types of compounds in ESYWLRHW

| No. | Metabolite name | m/z | Formula | CAS number | Ontology |
| --- | --- | --- | --- | --- | --- |
| 1 | D-Mannosamine | 202.0681348 | C_6_H_13_NO_5_ | 14307-02-9 | Carbohydrates |
| 2 | Susrose | 365.1055711 | C_12_H_22_O_11_ | 57-50-1 | Carbohydrates |
| 3 | Betaine | 118.0856785 | C_5_H_11_NO_2_ | 107-43-7 | Alkaloids |
| 4 | Maltotriose | 527.1590913 | C_18_H_32_O_16_ | 1109-28-0 | Carbohydrates |
| 5 | 2-Acetylacteoside | 689.2119719 | C_31_H_38_O_16_ | 94492-24-7 | Organic acid esters |
| 6 | Adenine | 136.061286 | C_5_H_5_N_5_ | 73-24-5 | Nucleobases |
| 7 | Guanine | 152.0564218 | C_5_H_5_N_5_O | 73-40-5 | Nucleobases |
| 8 | β-Gentiobiose | 360.1504655 | C_12_H_22_O_11_ | 5996-00-9 | Carbohydrates |
| 9 | Nicotinic acid | 124.0387616 | C_6_H_5_NO_2_ | 59-67-6 | Organic acids |
| 10 | Nicotinamide | 123.0550005 | C_6_H_6_N_2_O | 98-92-0 | Alkaloids |
| 11 | 5-Oxo-L-Proline | 130.0493715 | C_5_H_7_NO_3_ | 98-79-3 | Amino acids |
| 12 | Uridine | 245.0771138 | C_9_H_12_N_2_O_6_ | 58-96-8 | Nucleosides |
| 13 | (+/-) Salsolinol | 180.1012902 | C_10_H_13_NO_2_ | 525-72-4 | Alkaloids |
| 14 | Ac-Glu | 190.070549 | C_7_H_11_NO_5_ | 1188-37-0 | Organic acids |
| 15 | Tyrosine | 182.0807316 | C_18_H_32_O_7_ | 60-18-4 | Amino acids |
| 16 | Adenosine | 268.1041915 | C_10_H_13_N_5_O_4_ | 58-61-7 | Nucleosides |
| 17 | L-Phenylalanine | 166.0858941 | C_9_H_11_NO_2_ | 63-91-2 | Amino acids |
| 18 | 2-amino-5-[2-[[2,3-dihydroxy-2-(1-hydroxyethyl)butanoyl]oxymethyl]-4-hydroxyanilino]-5-oxopentanoic acid | 415.1715443 | C_18_H_26_N_2_O_9_ | NA | Organic acids |
| 19 | N-Caffeoylputrescine | 251.1394346 | C_13_H_18_N_2_O_3_ | 26148-06-1 | Alkaloids |
| 20 | 5-Caffeoylquinic acids | 355.1029461 | C_16_H_18_O_9_ | 1899964-29-4 | Organic acids |
| 21 | 2-amino-5-[2-[[(3S)-2,3-dihydroxy-2-[(1S)-1-hydroxyethyl]butanoyl]oxymethyl]anilino]-5-oxopentanoic acid | 399.1764616 | C_18_H_26_N_2_O_8_ | NA | Organic acids |
| 22 | 8-Hydroxyquinoline | 146.0588551 | C_9_H_7_NO | 148-24-3 | Alkaloids |
| 23 | L-Tryptophan | 205.0965028 | C_11_H_12_N_2_O_2_ | 73-22-3 | Amino acids |
| 24 | Norcoclaurine | 272.1280636 | C_16_H_17_NO_3_ | 22672-77-1 | Alkaloids |
| 25 | [3,4,5-trihydroxy-6-(3,4,5-trihydroxybenzoyl)oxyoxan-2-yl]methyl 3,4,5-trihydroxybenzoate | 502.1199571 | C_20_H_20_O_14_ | NA | Tannins |
| 26 | 2',5-digalloylhamamelofuranose | 507.0754386 | C_20_H_20_O_14_ | NA | Carbohydrates |
| 27 | Bergenin | 329.0870269 | C_14_H_16_O_9_ | 477-90-7 | Organic acid esters |
| 28 | 5,7-dihydroxy-2-methyl-8-[(2S,3R,4R,5S,6R)-3,4,5-trihydroxy-6-(hydroxymethyl)oxan-2-yl]chromen-4-one | 355.1020422 | C_16_H_18_O_9_ | 152041-16-2 | Phenolic glycosides |
| 29 | Syringin | 390.1761155 | C_17_H_24_O_9_ | 118-34-3 | Phenolic glycosides |
| 30 | 3-Formylindole | 146.0595974 | C_9_H_7_NO | 487-89-8 | Indoles |
| 31 | [(1S,21S,22R,23R)-6,7,8,11,12,13,22,23-octahydroxy-3,16-dioxo-2,17,20-trioxatetracyclo[17.3.1.04,9.010,15]tricosa-4,6,8,10,12,14-hexaen-21-yl] 3,4,5-trihydroxybenzoate | 652.1154266 | C_27_H_22_O_18_ | NA | Tannins |
| 32 | Corilagin | 657.0721914 | C_27_H_22_O_18_ | 23094-69-1 | Tannins |
| 33 | Vicenin II | 595.1662007 | C_27_H_30_O_15_ | 23666-13-9 | Flavonoids |
| 34 | Riboflavin | 377.1461236 | C_17_H_20_N_4_O_6_ | 83-88-5 | Vitamins |
| 35 | Quercetin 3,4′-diglucoside | 627.1560935 | C_27_H_30_O_17_ | 29125-80-2 | Flavonoid glycosides |
| 36 | Hippuric acids | 180.0650366 | C_9_H_9_NO_3_ | 495-69-2 | Organic acids |
| 37 | 4-[2-[[(3S)-2,3-dihydroxy-2-[(1S)-1-hydroxyethyl]butanoyl]oxymethyl]anilino]-4-oxobutanoic acid | 370.1501625 | C_17_H_23_NO_8_ | NA | Organic acids |
| 38 | 5,7-dihydroxy-2-(4-hydroxyphenyl)-8-[3,4,5-trihydroxy-6-(hydroxymethyl)oxan-2-yl]-6-(3,4,5-trihydroxyoxan-2-yl)chromen-4-one | 565.1552977 | C_26_H_28_O_14_ | 73543-87-0 | Flavonoid glycosides |
| 39 | Isoshaftoside | 587.1372216 | C_26_H_28_O_14_ | 52012-29-0 | Flavonoid glycosides |
| 40 | 3-[3,4-dihydroxy-6-(hydroxymethyl)-5-[3,4,5-trihydroxy-6-(hydroxymethyl)oxan-2-yl]oxyoxan-2-yl]oxy-2-(3,4-dihydroxyphenyl)-5,7-dihydroxychromen-4-one | 627.1567089 | C_27_H_30_O_17_ | NA | Flavonoid glycosides |
| 41 | 3,5,5-trimethyl-4-[[(2R,3R,4S,5S,6R)-3,4,5-trihydroxy-6-(hydroxymethyl)oxan-2-yl]oxymethyl]cyclohex-2-en-1-one | 331.1751079 | C_16_H_26_O_7_ | NA | Glycosides |
| 42 | Rutin | 611.1626348 | C_27_H_30_O_16_ | 153-18-4 | Flavonoid glycosides |
| 43 | 2-(3,4-dihydroxyphenyl)-5,7-dihydroxy-3-[(2S,3R,4S,5S,6R)-3,4,5-trihydroxy-6-[[(2S,3R,4S,5R)-3,4,5-trihydroxyoxan-2-yl]oxymethyl]oxan-2-yl]oxychromen-4-one | 597.1467384 | C_26_H_28_O_16_ | 142905-18-8 | Flavonoid glycosides |
| 44 | (2S,3S,4S,5R,6R)-6-(3-benzoyloxy-2-hydroxypropoxy)-3,4,5-trihydroxyoxane-2-carboxylic acid | 390.1401515 | C_16_H_20_O_10_ | NA | Organic acids |
| 45 | Violanthin | 579.1714337 | C_27_H_30_O_14_ | 40581-17-7 | Flavonoid glycosides |
| 46 | Panasenoside | 611.1627424 | C_27_H_30_O_16_ | 31512-06-8 | Flavonoid glycosides |
| 47 | Hyperoside | 465.1035816 | C_21_H_20_O_12_ | 482-36-0 | Flavonoid glycosides |
| 48 | Nepetin-7-glucoside | 479.1185143 | C_22_H_22_O_12_ | 569-90-4 | Flavonoid glycosides |
| 49 | Isorhamnetin | 317.0657415 | C_16_H_12_O_7_ | 480-19-3 | Flavonoids |
| 50 | 3-[(2S,3R,4S,5S,6R)-4,5-dihydroxy-6-(hydroxymethyl)-3-[(2S,3R,4S,5S,6R)-3,4,5-trihydroxy-6-(hydroxymethyl)oxan-2-yl]oxyoxan-2-yl]oxy-2-(3,4-dihydroxyphenyl)-5-hydroxy-7-methoxychromen-4-one | 641.1733964 | C_28_H_32_O_17_ | 259234-17-8 | Flavonoid glycosides |
| 51 | Quercetin-3,7-O-a-L-dirhamnoside | 595.1671541 | C_27_H_30_O_15_ | NA | Flavonoid glycosides |
| 52 | Liquiritin apioside | 551.1761421 | C_26_H_30_O_13_ | 74639-14-8 | Flavonoid glycosides |
| 53 | Vitexin | 433.1136128 | C_21_H_20_O_10_ | 3681-93-4 | Flavonoid glycosides |
| 54 | Isoquercetin | 465.1042229 | C_21_H_20_O_12_ | 482-35-9 | Flavonoid glycosides |
| 55 | peonidin-3-o-beta-d-glucopyranoside | 463.1243388 | C_22_H_23_O_11_ | NA | Flavonoid glycosides |
| 56 | Kaempferol-3-Glucoside-3''-Rhamnoside | 595.1672469 | C_27_H_30_O_15_ | NA | Flavonoid glycosides |
| 57 | Ambocin | 565.1558066 | C_26_H_28_O_14_ | 108044-05-9 | Flavonoid glycosides |
| 58 | Isorhamnetin-3-O-rutinoside | 625.1777973 | C_28_H_32_O_16_ | 604-80-8 | Flavonoid glycosides |
| 59 | Apigenin 7-O-rutinoside | 579.1718768 | C_27_H_30_O_14_ | 552-57-8 | Flavonoid glycosides |
| 60 | 3-(3,4-dimethoxyphenyl)-5-hydroxy-10-(3-isopropoxy-4-methoxyphenyl)-9,10-dihydropyrano[2,3-f]chromene-4,8-dione | 533.1644331 | C_30_H_28_O_9_ | NA | Flavonoids |
| 61 | Diosmetin | 301.0703384 | C_16_H_12_O_6_ | 520-34-3 | Flavonoids |
| 62 | Peonidin-3-O-glucoside | 463.1237196 | C_22_H_23_O_11_ | 6906-39-4 | Flavonoid glycosides |
| 63 | Naringenin | 273.0757017 | C_15_H_12_O_5_ | 480-41-1 | Flavonoids |
| 64 | Vanillin | 153.0550896 | C_8_H_8_O_3_ | 121-33-5 | Phenols |
| 65 | Kaempferol | 287.0552958 | C_15_H_10_O_6_ | 520-18-3 | Flavonoids |
| 66 | Luteolin 7-O-glucoside | 449.1090449 | C_21_H_20_O_11_ | 1268798 | Flavonoid glycosides |
| 67 | Neodiosmin | 609.1833131 | C_28_H_32_O_15_ | 38665-01-9 | Flavonoid glycosides |
| 68 | Isorhamnetin-3-O-glucoside | 479.11829 | C_22_H_22_O_12_ | 5041-82-7 | Flavonoid glycosides |
| 69 | Baicalin | 447.0957471 | C_21_H_18_O_11_ | 21967-41-9 | Flavonoid glycosides |
| 70 | 3,5-Dicaffeoylquininic acid | 517.134865 | C_25_H_24_O_12_ | 89919-62-0 | Organic acids |
| 71 | Imperialine | 430.3317425 | C_27_H_43_NO_3_ | 61825-98-7 | Alkaloids |
| 72 | Diosmetin 7-O-β-D-glucuronide | 477.1045552 | C_22_H_20_O_12_ | 35110-20-4 | Flavonoid glycosides |
| 73 | Cryptopine | 370.1661782 | C_21_H_23_NO_5_ | 482-74-6 | Alkaloids |
| 74 | Isoliquiritin | 419.1337857 | C_21_H_22_O_9_ | 5041-81-6 | Flavonoid glycosides |
| 75 | Formononetin 7-O-β-D-apiofuranosyl-(1→6)-β-D-glucopyranoside | 563.1756327 | C_27_H_30_O_13_ | 857677-78-2 | Flavonoid glycosides |
| 76 | Scutellarein 4'-methyl ether | 301.070174 | C_16_H_12_O_6_ | 6563-66-2 | Flavonoid glycosides |
| 77 | [3,4,5-trihydroxy-6-[3-(4-methoxyphenyl)-4-oxochromen-7-yl]oxyoxan-2-yl]methyl 2-piperidin-4-ylacetate | 556.2190594 | C_29_H_33_NO_10_ | NA | Flavonoid glycosides |
| 78 | Formononetin 7-O-glucoside | 431.1338316 | C_22_H_22_O_9_ | 486-62-4 | Flavonoid glycosides |
| 79 | Altenuene | 315.0706438 | C_15_H_16_O_6_ | 29752-43-0 | Organic acid esters |
| 80 | Andrographidin B | 493.1341464 | C_23_H_24_O_12_ | 113963-38-5 | Flavonoid glycosides |
| 81 | Nepetin | 317.0654006 | C_16_H_12_O_7_ | 520-11-6 | Flavonoids |
| 82 | Cirsimarin | 477.1398453 | C_23_H_24_O_11_ | 13020-19-4 | Flavonoids |
| 83 | Paprazine | 284.127253 | C_17_H_17_NO_3_ | 36417-86-4 | Phenylpropanoids |
| 84 | Licochalcone B | 287.0915372 | C_16_H_14_O_5_ | 58749-23-8 | Flavonoids |
| 85 | Daidzein | 255.0655133 | C_15_H_10_O_4_ | 486-66-8 | Flavonoids |
| 86 | N-trans-Feruloyltyramine | 314.1388798 | C_18_H_19_NO_4_ | 66648-43-9 | Alkaloids |
| 87 | (±)-Liquiritigenin | 257.0809147 | C_15_H_12_O_4_ | 69097-97-8 | Flavonoids |
| 88 | Luteolin | 287.055071 | C_15_H_10_O_6_ | 491-70-3 | Flavonoids |
| 89 | calycosin | 285.0753291 | C_16_H_12_O_5_ | 20575-57-9 | Flavonoids |
| 90 | Dicoumaroyl Spermidine | 438.2401079 | C_25_H_31_N_3_O_4_ | 65715-79-9 | Alkaloids |
| 91 | Hispidulin | 301.070695 | C_16_H_12_O_6_ | 1447-88-7 | Flavonoids |
| 92 | Hexahydrocurcumin | 375.1791797 | C_21_H_26_O_6_ | 36062-05-2 | Carbohydrates |
| 93 | Licoricesaponin G2 | 839.4070368 | C_42_H_62_O_17_ | 118441-84-2 | Triterpene saponins |
| 94 | Apigenin | 271.0605973 | C_15_H_10_O_5_ | 520-36-5 | Flavonoids |
| 95 | Tricin | 331.0818323 | C_17_H_14_O_7_ | 520-32-1 | Flavonoids |
| 96 | Chrysoeriol | 301.0710809 | C_16_H_12_O_6_ | 491-71-4 | Flavonoids |
| 97 | [5-acetyloxy-1,7-bis(3,4-dihydroxyphenyl)heptan-3-yl] acetate | 450.2123426 | C_23_H_28_O_8_ | 915039-08-6 | Organic acid esters |
| 98 | (Z)-5,8,11-trihydroxyoctadec-9-enoic acid | 348.2746957 | C_18_H_34_O_5_ | NA | Carboxylic acids |
| 99 | Cirsiliol | 331.0808819 | C_17_H_14_O_7_ | 34334-69-5 | Flavonoids |
| 100 | Licoricesaponin H2 | 823.4115542 | C_42_H_62_O_16_ | 118441-85-3 | Triterpene saponins |
| 101 | glycyrrhetic acid | 471.3482171 | C_30_H_46_O_4_ | 471-53-4 | Terpenes |
| 102 | (3beta,20beta)-29-hydroxy-11,29-dioxoolean-12-en-3-yl 2-O-beta-D-glucopyranuronosyl-alpha-D-glucopyranosiduronic acid | 845.3930645 | C_42_H_62_O_16_ | NA | Triterpene saponins |
| 103 | Liquiritigenin | 257.0814777 | C_15_H_12_O_4_ | 578-86-9 | Flavonoids |
| 104 | (2S,3S,4S,5R,6R)-6-[(2R,3R,4S,5S,6S)-2-[[(3S,6aR,6bS,8aS,12aR,14bS)-11-carboxy-4,4,6a,6b,8a,11,14b-heptamethyl-14-oxo-2,3,4a,5,6,7,8,9,10,12,12a,14a-dodecahydro-1H-picen-3-yl]oxy]-6-carboxy-4,5-dihydroxyoxan-3-yl]oxy-3,4,5-trihydroxyoxane-2-carboxylic acid | 823.4118377 | C_42_H_62_O_16_ | NA | Triterpene saponins |
| 105 | Glycyrrhizic acid | 845.3946173 | C_42_H_62_O_16_ | 1405-86-3 | Triterpene saponins |
| 106 | 4',7-Dimethoxy-3-hydroxyflavone | 299.0922461 | C_17_H_14_O_5_ | NA | Flavonoids |
| 107 | Cirsimaritin | 315.0861915 | C_17_H_14_O_6_ | 6601-62-3 | Flavonoids |
| 108 | Aurantiamide | 403.201999 | C_25_H_26_N_2_O_3_ | 58115-31-4 | Alkaloids |
| 109 | Piperyline | 272.1282369 | C_16_H_17_NO_3_ | 25924-78-1 | Alkaloids |
| 110 | 2-[(1S,2S,4aR,8aS)-1-Hydroxy-4a-methyl-8-methylenedecahydro-2-naphthalenyl]acrylic acid | 233.1534408 | C_15_H_22_O_3_ | NA | Terpenes |
| 111 | (5R)-5-hydroxy-1-(4-hydroxy-3-methoxyphenyl)decan-3-one | 277.1795465 | C_17_H_26_O_4_ | 72749-01-0 | Phenols |
| 112 | 6-Gingerol | 295.1911806 | C_17_H_26_O_4_ | 23513-14-6 | Phenols |
| 113 | Ganoderic Acid H | 573.3045653 | C_32_H_44_O_9_ | 98665-19-1 | Terpenes |
| 114 | Piperanine | 288.1590304 | C_17_H_21_NO_3_ | 23512-46-1 | Alkaloids |
| 115 | Piperine | 286.1429622 | C_17_H_19_NO_3_ | 94-62-2 | Alkaloids |
| 116 | Eupatilin | 345.0974279 | C_18_H_16_O_7_ | 22368-21-4 | Flavonoids |
| 117 | Aurantiamide acetate | 445.2127281 | C_27_H_28_N_2_O_4_ | 56121-42-7 | Alkaloids |
| 118 | 5,7-Dimethoxy-4-methylcoumarin | 221.0806465 | C_12_H_12_O_4_ | 6093-80-7 | Phenylpropanoids |
| 119 | Niranthin | 415.2116017 | C_24_H_32_O_7_ | 50656-77-4 | Phenylpropanoids |
| 120 | Methylxanthoxylin | 211.0962824 | C_11_H_14_O_4_ | 23121-32-6 | Carbohydrates |
| 121 | 4-(8-prop-1-en-2-yl-3,4,8,9-tetrahydro-2H-furo[2,3-h]chromen-3-yl)benzene-1,3-diol | 325.1442073 | C_20_H_20_O_4_ | NA | Flavonoids |
| 122 | 5-Demethylnobiletin | 389.1227871 | C_20_H_20_O_8_ | 2174-59-6 | Flavonoids |
| 123 | Glabrol | 393.2061798 | C_25_H_28_O_4_ | 59870-65-4 | Flavonoids |
| 124 | 6-Shogaol | 277.1794653 | C_17_H_24_O_3_ | 555-66-8 | Phenols |
| 125 | 6-Hydroxy-5a-methyl-3,9-bis(methylene)decahydronaphtho[1,2-b]furan-2(3H)-one | 231.1375208 | C_15_H_20_O_3_ | 29424-04-2 | Organic acid esters |
| 126 | 2,2'-(Tetradecylimino)diethanol | 302.3064838 | C_18_H_39_NO_2_ | 18924-66-8 | Alkaloids |
| 127 | Pipernonaline | 342.2060743 | C_21_H_27_NO_3_ | 88660-10-0 | Alkaloids |
| 128 | Humulene | 205.1950175 | C_15_H_24_ | 6753-98-6 | Terpenes |
| 129 | Piperoleine B | 344.2224277 | C_21_H_29_NO_3_ | 30505-89-6 | Alkaloids |
| 130 | Dibutylphthalate | 279.1589182 | C_16_H_22_O_4_ | 84-74-2 | Organic acid esters |
| 131 | 8-Shogaol | 305.2113092 | C_19_H_28_O_3_ | 36700-45-5 | Phenols |
| 132 | Guineensine | 384.2531637 | C_24_H_33_NO_3_ | 55038-30-7 | Alkaloids |
| 133 | Acetyl tributyl citrate | 403.2316008 | C_20_H_34_O_8_ | 77-90-7 | Organic acid esters |
| 134 | 10-Shogaol | 333.2423312 | C_21_H_32_O_3_ | 36752-54-2 | Phenols |
| 135 | Dihydroergotamine | 584.2809614 | C_33_H_37_N_5_O_5_ | 511-12-6 | Alkaloids |
| 136 | Brachystamide B | 412.2849505 | C_26_H_37_NO_3_ | 126394-65-8 | Alkaloids |
| 137 | Fortunellin | 593.193849 | C_28_H_32_O_14_ | 20633-93-6 | Flavonoid glycosides |
| 138 | (2E,4E,12Z)-N-(2-methylpropyl)octadeca-2,4,12-trienamide | 334.3093592 | C_22_H_39_NO | 151391-69-4 | Alkaloids |
| 139 | Dioctylphthalate | 391.28382 | C_24_H_38_O_4_ | 117-81-7；82208-43-3 | Organic acid esters |
| 140 | D-Glucopyranose | 179.0559848 | C_6_H_12_O_6_ | 2280-44-6 | Carbohydrates |
| 141 | (-)-Quinic acid | 191.0560646 | C_7_H_12_O_6_ | 77-95-2 | Organic acids |
| 142 | Shikimic Acid | 173.04554 | C_7_H_10_O_5_ | 138-59-0 | Organic acids |
| 143 | N-Acetyl-L-glutamic acid | 188.0562963 | C_7_H_11_NO_5_ | 1188-37-0 | Amino acids |
| 144 | Citrate | 191.0198477 | C_6_H_8_O_7_ | 126-44-3 | Carboxylic acids |
| 145 | Guanosine | 282.0840609 | C_10_H_13_N_5_O_5_ | 118-00-3 | Nucleosides |
| 146 | Isoleucine | 130.0872184 | C_6_H_13_NO_2_ | 73-32-5 | Amino acids |
| 147 | Gallic acid | 169.0138513 | C_7_H_6_O_5_ | 149-91-7 | Organic acids |
| 148 | Protocatechuic acid | 153.0192425 | C_7_H_6_O_4_ | 99-50-3 | Organic acids |
| 149 | Neochlorogenic acid | 353.0873214 | C_16_H_18_O_9_ | 906-33-2 | Organic acids |
| 150 | Citrazinc acid | 154.0140424 | C_6_H_5_NO_4_ | 99-11-6 | Organic acids |
| 151 | α-Isopropylmalate | 175.0605675 | C_7_H_12_O_5_ | 3237-44-3 | Carboxylic acids |
| 152 | o-Hydroxybenzoate | 137.0244716 | C_7_H_6_O_3_ | 63-36-5 | Organic acids |
| 153 | Flavonol base + 4O, O-Hex-Hex, O-dHex | 771.1978594 | C_33_H_40_O_21_ | NA | Flavonoids |
| 154 | Pimelic acid | 159.0662158 | C_7_H_12_O_4_ | 111-16-0 | Carboxylic acids |
| 155 | (3R,4S,5R)-4-hydroxy-3,5-bis[(3,4,5-trihydroxybenzoyl)oxy]cyclohexene-1-carboxylic acid | 477.0669832 | C_21_H_18_O_13_ | 95753-52-9 | Organic acid esters |
| 156 | Hydrangeifolin I | 461.1664244 | C_19_H_28_O_10_ | 88510-10-5 | Glycosides |
| 157 | 1,3,6-Tri-O-galloyl-β-D-glucose | 635.0887089 | C_27_H_24_O_18_ | 18483-17-5 | Tannins |
| 158 | Isoorientin | 447.0932503 | C_21_H_20_O_11_ | 4261-42-1 | Flavonoid glycosides |
| 159 | Kaempferol 3-O-sophoroside | 609.1454399 | C_27_H_30_O_16_ | 19895-95-5 | Flavonoid glycosides |
| 160 | 5,7-dihydroxy-2-(4-hydroxyphenyl)-6,8-bis(3,4,5-trihydroxyoxan-2-yl)chromen-4-one | 533.1288613 | C_25_H_26_O_13_ | NA | Flavonoid glycosides |
| 161 | 1,2,3,6-Tetra-O-galloyl-β-D-glucose | 787.0990095 | C_34_H_28_O_22_ | 79886-50-3 | Tannins |
| 162 | Prunin | 433.113771 | C_21_H_22_O_10_ | 529-55-5 | Flavonoid glycosides |
| 163 | Ellagic Acid | 300.998764 | C_14_H_6_O_8_ | 476-66-4 | Tannins |
| 164 | 7-hydroxy-2-[4-[(2S,3R,4S,5S,6R)-3,4,5-trihydroxy-6-(hydroxymethyl)oxan-2-yl]oxyphenyl]-2,3-dihydrochromen-4-one | 417.1188424 | C_21_H_22_O_9_ | NA | Flavonoids |
| 165 | trans-4-Hydroxycinnamic acid | 163.0400806 | C_9_H_8_O_3_ | 501-98-4 | Organic acids |
| 166 | Kaempferol-3-O-rutinoside | 593.150734 | C_27_H_30_O_15_ | 17650-84-9 | Flavonoid glycosides |
| 167 | 5,7-dihydroxy-2-(4-hydroxy-3-methoxyphenyl)-3-[3,4,5-trihydroxy-6-[[(2R,3R,4R,5R,6S)-3,4,5-trihydroxy-6-methyloxan-2-yl]oxymethyl]oxan-2-yl]oxychromen-4-one | 623.1608045 | C_28_H_32_O_16_ | NA | Flavonoids |
| 168 | 4,5-Dicaffeoylquinic acids | 515.119382 | C_25_H_24_O_12_ | 57378-72-0 | Organic acids |
| 169 | Quercetin-3-O-alpha-L-rhamnopyranoside | 447.0926914 | C_21_H_20_O_11_ | NA | Flavonoid glycosides |
| 170 | 4-O-Caffeoylquinic acid | 353.0878016 | C_16_H_18_O_9_ | 905-99-7 | Organic acids |
| 171 | Aspirin | 179.0346654 | C_9_H_8_O_4_ | 50-78-2 | Organic acids |
| 172 | 3-[(1-Carboxyvinyl)oxy]benzoic acid | 207.0295089 | C_10_H_8_O_5_ | 16929-37-6 | Organic acids |
| 173 | Glucoliquiritin apioside | 757.1990354 | C_32_H_40_O_18_ | 157226-47-6 | Flavonoid glycosides |
| 174 | Isorhamnetin 3-O-robinoside | 623.1408365 | C_28_H_32_O_16_ | 107740-46-5 | Flavonoid glycosides |
| 175 | Rosmarinic acid | 359.0768007 | C_18_H_16_O_8_ | 20283-92-5 | Organic acids |
| 176 | Azelaic acid | 187.0974281 | C_9_H_16_O_4_ | 123-99-9 | Carboxylic acids |
| 177 | Salvianolic acid B | 717.1446574 | C_36_H_30_O_16_ | 121521-90-2 | Flavonoids |
| 178 | NCGC00380479-01_C36H58O11_beta-D-Glucopyranose, 1-O-[(2alpha,3beta,5xi,6beta,9xi,19alpha)-2,3,6,19-tetrahydroxy-28-oxoolean-12-en-28-yl]- | 711.394768 | C_36_H_58_O_11_ | NA | Terpenes |
| 179 | Secoisolariciresinol | 361.1651348 | C_20_H_26_O_6_ | 29388-59-8 | Phenylpropanoids |
| 180 | Tricin 5-O-β-D-glucopyranoside | 491.1192431 | C_23_H_24_O_12_ | 32769-00-9 | Flavonoid glycosides |
| 181 | Rhamnetin 3-galactoside | 477.1035167 | C_22_H_22_O_12_ | 62858-07-5 | Flavonoid glycosides |
| 182 | NCGC00385736-01 | 823.4084982 | C_42_H_64_O_16_ | NA | Triterpene saponins |
| 183 | β-D-Glucopyranose, 6-[(2E)-3-phenyl-2-propenoate] 1-(3,4,5-trihydroxybenzoate) | 461.1078013 | C_22_H_22_O_11_ | 115746-69-5 | Tannins |
| 184 | Quercetin | 301.0348331 | C_15_H_10_O_7_ | 117-39-5 | Flavonoids |
| 185 | Chebuloside II | 711.3962951 | C_36_H_58_O_11_ | 149475-28-5 | Triterpene saponins |
| 186 | 6-[[8a-carboxy-4,4,6a,6b,11,11,14b-heptamethyl-10-[3,4,5-trihydroxy-6-(hydroxymethyl)oxan-2-yl]oxy-1,2,3,4a,5,6,9,10,12,12a,14,14a-dodecahydropicen-3-yl]oxy]-3,4-dihydroxy-5-[3,4,5-trihydroxy-6-(hydroxymethyl)oxan-2-yl]oxyoxane-2-carboxylic acid | 969.4678477 | C_48_H_74_O_20_ | NA | Terpenes |
| 187 | Prunetin | 283.0609522 | C_16_H_12_O_5_ | 552-59-0 | Flavonoids |
| 188 | (-)-Fulgidic acid | 327.2174219 | C_18_H_32_O_5_ | 95341-44-9 | Carboxylic acids |
| 189 | Aloe-emodin | 269.045257 | C_15_H_10_O_5_ | 481-72-1 | Anthraquinones |
| 190 | Naringenin chalcone | 271.0610607 | C_15_H_12_O_5_ | 25515-46-2 | Flavonoids |
| 191 | Enterolactone | 297.1116162 | C_18_H_18_O_4_ | 78473-71-9 | Phenylpropanoids |
| 192 | Isoliquiritigenin | 255.0656857 | C_15_H_12_O_4_ | 961-29-5 | Flavonoids |
| 193 | (1S,4aR,6aS,6bR,9R,10R,11R,12aR,14bS)-1,10,11-trihydroxy-9-(hydroxymethyl)-2,2,6a,6b,9,12a-hexamethyl-1,3,4,5,6,6a,7,8,8a,10,11,12,13,14b-tetradecahydropicene-4a-carboxylic acid | 503.337088 | C_30_H_48_O_6_ | NA | Terpenes |
| 194 | Formononetin | 267.0659631 | C_16_H_12_O_4_ | 485-72-3 | Flavonoids |
| 195 | isokaempferide | 299.054992 | C_16_H_12_O_6_ | 1592-70-7 | Flavonoids |
| 196 | Warfarin | 307.0954933 | C_19_H_16_O_4_ | 81-81-2 | Flavonoids |
| 197 | Spiculisporic acid | 327.1806014 | C_17_H_28_O_6_ | 469-77-2 | Carboxylic acids |
| 198 | Hexosyl LPE 18:2 | 638.3284291 | C_29_H_54_NO_12_P | NA | Monosaccharide phosphates |
| 199 | Arjunic acid | 487.3413445 | C_30_H_48_O_5_ | 31298-06-3 | Terpenes |
| 200 | 9,10-DiHOME | 313.2374624 | C_18_H_34_O_4_ | 263399-34-4 | Carboxylic acids |
| 201 | Hexosyl LPE 16:0 | 614.3271134 | C_27_H_54_NO_12_P | NA | Monosaccharide phosphates |
| 202 | (3S,4S,6aR,6bS,8R,8aR,12aS,14bR)-8-hydroxy-4,6a,6b,11,11,14b-hexamethyl-3-[(2S,3R,4S,5R)-3,4,5-trihydroxyoxan-2-yl]oxy-1,2,3,4a,5,6,7,8,9,10,12,12a,14,14a-tetradecahydropicene-4,8a-dicarboxylic acid | 633.3759574 | C_35_H_54_O_10_ | NA | Terpenes |
| 203 | Panaxcerol B | 559.3121946 | C_27_H_46_O_9_ | 171520-42-6 | Organic acid esters |
| 204 | 13-HOTrE | 293.2115122 | C_18_H_30_O_3_ | 87984-82-5 | Carboxylic acids |
| 205 | 12,13-Epoxy-9-octadecenoic acid | 295.2267371 | C_18_H_32_O_3_ | 6799-85-5 | Carboxylic acids |
| 206 | Maslinic acid | 517.3525054 | C_30_H_48_O_4_ | 4373-41-5 | Terpenes |
